# Navigation and Sensor Fusion for Autonomous Field Robots in Precision Agriculture: Narrative Review

**DOI:** 10.3390/s26165169

**Published:** 2026-08-15

**Authors:** Norbert Boros, Bálint Ambrus, Anikó Nyéki

**Affiliations:** Department of Biosystems and Precision Technology, Albert Kázmér Faculty of Mosonmagyaróvár, Széchenyi István University, 9200 Mosonmagyaróvár, Hungary; boros.norbert@sze.hu (N.B.); nyeki.aniko@sze.hu (A.N.)

**Keywords:** precision agriculture, field robot, autonomous navigation, sensor fusion, GNSS/IMU, SLAM, ROS 2

## Abstract

Autonomous field robots are increasingly used in precision agriculture for monitoring, phenotyping, spraying, and site-specific intervention, yet reliable autonomy remains difficult under vegetation occlusion, uneven terrain, variable illumination, and intermittent communications. This review provides a deployment-oriented synthesis of navigation and sensor-fusion methods for agricultural robots, with emphasis on what is practical under field conditions rather than only in laboratory settings. The literature was examined through a structured narrative-review workflow using Scopus, Web of Science, IEEE Xplore, ScienceDirect, SpringerLink, and related citation tracking, with primary emphasis on studies published between 2015 and 2025. We compare global, local, and hybrid planning methods; motion-control strategies such as PID, Pure Pursuit, and MPC; and localization pipelines that combine GNSS, IMU, LiDAR, cameras, odometry, and SLAM or Kalman-family fusion. Beyond algorithm summaries, the review links method selection to agricultural deployment constraints, including GNSS degradation, dynamic obstacles, compute limits, ROS 2 integration, time synchronization, and coordinate-frame management. The synthesis shows that no single stack is optimal across all crop systems: lightweight GNSS/IMU-based solutions remain attractive in structured open fields, whereas orchards, vineyards, and other occluded environments benefit more from tighter multi-sensor fusion and SLAM-supported localization. Finally, the review distills design guidance for sensing, planning, validation, and digital-twin-supported testing, and identifies research gaps related to robustness, benchmarking, safety, and scalable on-farm deployment.

## 1. Introduction

Agriculture is entering a period shaped by multiple, interrelated challenges. Climate change poses a serious threat to global food security; it can reduce the yields of major crops, while droughts, heatwaves, and floods further undermine productivity. Closely related is the problem of soil degradation; over the past 40 years, soil erosion, nutrient depletion, and the loss of organic matter have contributed to the loss of approximately 33% of arable land. Water scarcity has also become a critical constraint due to excessive water extraction. Declining biodiversity further weakens the resilience of agricultural systems, as monocultures and intensive chemical inputs suppress ecosystems—reducing adaptive capacity and jeopardizing natural processes such as pollination and the maintenance of soil fertility. Population growth and excessive food waste impose additional pressure on agricultural production. Urbanization constitutes another major challenge, as rapid city expansion converts fertile agricultural land into built environments. In addition, persistent labor shortages increasingly constrain the sector [1].

The goal of precision agriculture is to enable data-driven decision-making and the implementation of targeted interventions. Its foundation is continuous data collection using diverse sensors, which can be used to determine the biological and chemical parameters of crops. Continuous measurements allow the generation of spatial and temporal maps, from which the presence of diseases and pests can be inferred. Soil condition and nutrient content are likewise assessed using sensor-based measurements. Real-time sensing enables immediate intervention when required. In addition to disease monitoring, yield can also be tracked, supporting yield estimation. Based on crop status, targeted and optimized irrigation and spraying can be implemented through variable-rate application of nutrients and plant-protection products [2].

To perform such precision operations, precision mobile robots have emerged. These platforms carry out a range of tasks, including data acquisition, weeding, crop protection, and harvesting, as well as various combinations of these functions [3].

**Figure 1:**
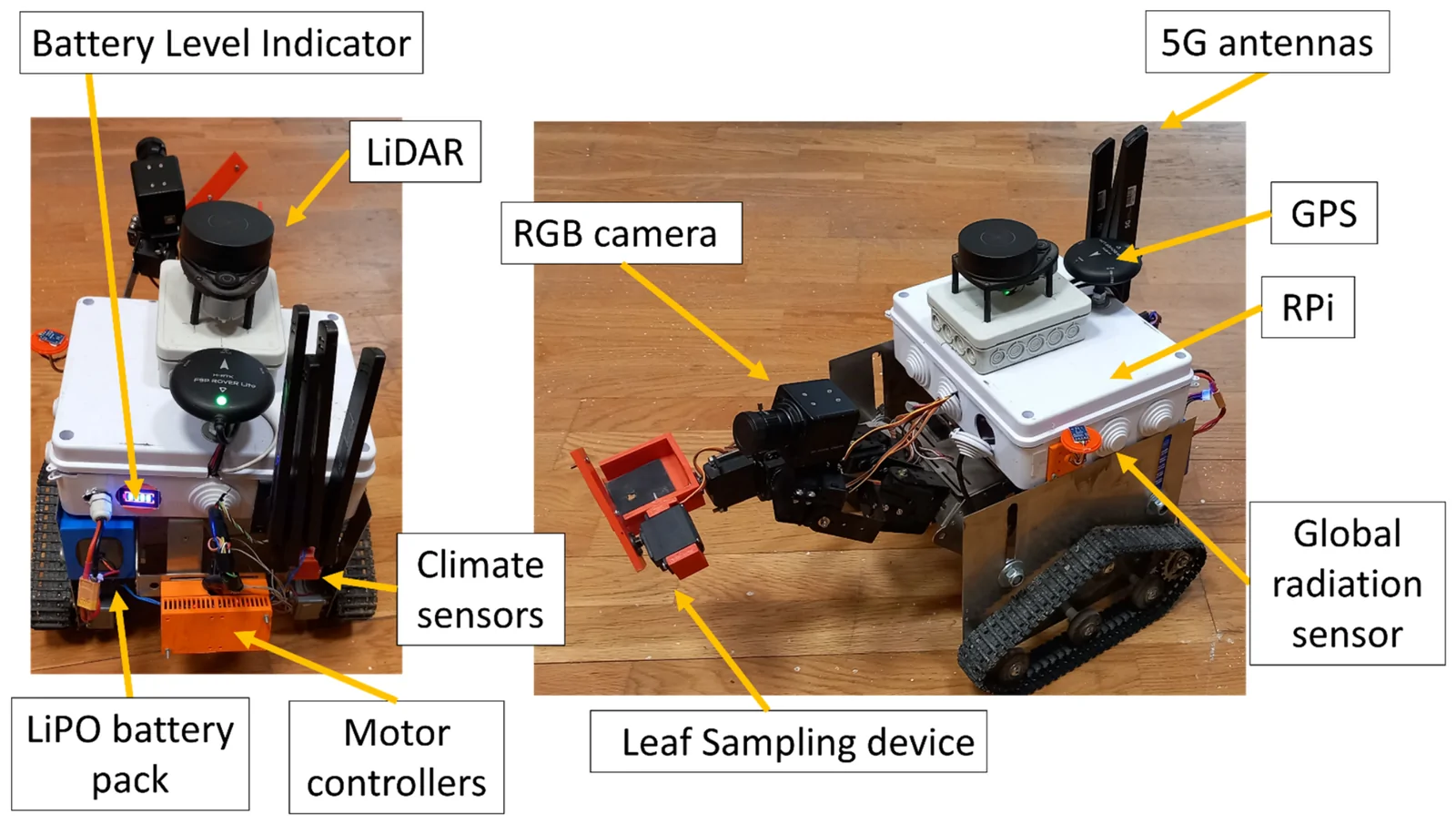
Monitoring Mobile Robot [3].

The precision monitoring mobile robot developed by Ambrus (Figure 1) performs environmental sensing using LiDAR, an RGB camera, ultrasonic sensors, global radiation measurement, and soil sensors.

Agricultural robots can be used for spraying, fertilization, soil-condition assessment, weeding, and the collection of crop phenotypic traits. Modern systems are also capable of identifying individual plants and applying targeted treatments, and they increasingly support automated harvesting as well as selective crop management [4].

This review develops a deployment-oriented synthesis of navigation and sensor-fusion solutions for autonomous field robots in precision agriculture. It distinguishes between general-purpose robotics methods and approaches validated in, or directly transferable to, agricultural environments, and it links algorithm choice to field constraints such as vegetation occlusion, uneven terrain, variable illumination, and intermittent connectivity. In addition to planning, control, and localization methods, the review addresses integration issues that often determine real-world performance, including time synchronization, ROS 2/DDS communication, TF management, edge–cloud computing, and digital-twin-supported testing. The aim is not only to summarize methods, but to extract design guidance for selecting sensing, planning, and control stacks that are realistic for on-farm deployment.

## 2. Methodology

The aim of this review is to synthesize navigation and sensor-fusion solutions for autonomous field robots in precision agriculture from a deployment-oriented perspective. Because the topic spans robotics, control, sensing, and agricultural engineering, the literature was examined at two complementary levels: (i) general mobile-robotics methods and (ii) approaches that have been validated in, or can be transferred with minimal adaptation to, agricultural environments.

This article is a narrative review informed by a structured literature search rather than a formal systematic review or meta-analysis. Primary searches were conducted in Scopus, Web of Science, IEEE Xplore, ScienceDirect, SpringerLink, and MDPI. Google Scholar was used as a supplementary tool for backward and forward citation tracking and for identifying additional peer-reviewed studies relevant to field robotics.

The core search logic combined crop-domain terms (e.g., ‘agricultural robot’, ‘field robot’, and ‘precision agriculture’) with navigation and autonomy terms (e.g., ‘navigation’, ‘localization’, ‘path planning’, ‘trajectory tracking’, ‘SLAM’, ‘sensor fusion’, ‘GNSS’, ‘IMU’, ‘LiDAR’, ‘camera’, ‘ROS 2’, ‘MPC’, ‘DWA’, and ‘TEB’). The main review window covered 2015–2025, while older studies were retained selectively when they provided foundational theory, canonical algorithms, or standards still used in current systems.

Table 1 summarizes the total number of search results returned for the primary search term in different scientific databases, illustrating the literature coverage of each database.

Table 2 presents the annual percentage distribution of the combined search results from the scientific databases, illustrating the temporal trends in publication activity within the investigated research topics.

The search results indicate a significant increase in scientific interest in the investigated research topics over recent years. The growing number of publications suggests that research on precision mobile robots and precision agriculture is currently a rapidly evolving and increasingly important field of study.

Studies were prioritized when they (i) addressed localization, mapping, planning, control, or sensor fusion for mobile robots, (ii) reported sufficient methodological detail to support interpretation, and (iii) included evidence from field trials, experiments, simulations, or realistic implementation studies. Papers focused solely on highly structured industrial settings, with no clear transfer path to agricultural deployment, were excluded from the main synthesis. The final narrative synthesis emphasizes practical trade-offs among sensing stack, computational burden, robustness to field disturbances, and integration requirements.

## 3. Autonomous Mobile Platforms in Precision Crop Production

Mobile robots are mechanical systems capable of autonomous movement within their environment. They are deployed across a wide range of target tasks and research domains. For reliable operation, they must be equipped with sensors, actuators, and embedded intelligence—i.e., control algorithms [5,6].

Autonomous mobile robots are a class of mobile robots that are capable of fully independent navigation. These robots are equipped with various sensors and cameras that enable them to perceive and interpret their surroundings. As a result, they can localize themselves in the environment and avoid obstacles in order to execute their target tasks accurately [7,8].

To address challenges related to productivity, environmental impacts, food security, yield losses, and sustainability, autonomous mobile robots are increasingly being adopted in precision agriculture. These systems primarily integrate crop information acquisition processes, thereby contributing to improving the efficiency of agricultural production [9].

### 3.1. Challenges for Robots in Agricultural Environments

Agricultural environments pose numerous challenges that are less common in urban settings and on paved roads. Wheel slip on loose soil, mud, or wet grass reduces the accuracy of wheel odometry, leading to accumulating localization errors. Sloped and uneven terrain alters vehicle dynamics and may increase path-tracking error. Soil sinkage also changes the vehicle’s actual motion, further reducing path-tracking accuracy.

Unlike road environments, agricultural areas generally lack permanent infrastructure elements such as road markings, lane markings, traffic signs, or readily identifiable artificial landmarks that could serve as reliable reference points for localization. Instead, navigation must rely largely on crop rows and features of the natural environment, both of which change continuously.

Crop growth stage, weed pressure, changing illumination, shadows, missing or curved crop rows, and wheel tracks can all affect the performance of camera- and LiDAR-based perception systems. Consequently, the effectiveness of conventional vision-based algorithms may deteriorate substantially under such conditions [10,11].

When designing agricultural robots, it is important to determine whether they are intended for open-field or greenhouse operation. Greenhouse robots must cope with GNSS-denied localization and narrow, continuously changing workspaces that are often shared with human workers. In addition, the robot must be able to operate under loose-soil conditions, high temperatures, and high humidity [12].

### 3.2. Platform Types

Based on their locomotion mode, mobile robot platforms can be classified into two main groups: wheeled and tracked systems. Although legged (walking) robots also exist, in practice wheeled and tracked designs are the most widespread; therefore, the remainder of this section focuses on these two categories.

The comparison between the two types is particularly relevant in terms of mobility (degrees of freedom) and travel speed. In addition to total vehicle mass, performance is influenced by factors such as ground pressure (i.e., the pressure exerted by tires or tracks) and traction characteristics. Tests conducted on different soil and obstacle types indicate that there is no universally optimal solution; rather, both systems have distinct advantages and disadvantages.

A key advantage of wheeled vehicles is their performance on paved surfaces or well-prepared firm terrain, where they can provide higher speed, faster progress, and more precise motion during operations on relatively flat ground. In contrast, tracked vehicles tend to perform better under uneven, loose soil conditions, where they can maintain more stable motion than wheeled solutions. A further advantage of wheeled vehicles is that they are typically more economical to operate than tracked platforms [13,14].

In agricultural applications, due to the structural characteristics of soil, tracked propulsion is often the more suitable option; however, wheeled systems generally provide higher precision and controllability. Therefore, a range of studies has focused on improving tracked platforms and on combining the two propulsion modes.

One approach is the development of a new three-dimensional dynamic model for agricultural tracked vehicles, describing platform motion with eight degrees of freedom. This model can significantly improve maneuverability and motion accuracy under varying terrain conditions [15].

Another direction is the design of hybrid solutions that integrate the advantages of platforms with different propulsion modes. Several hybrid concepts have been proposed. In one design, conventional wheels are integrated alongside tracks, allowing the robot to select the locomotion mode best suited to the current surface [16].

**Figure 2:**
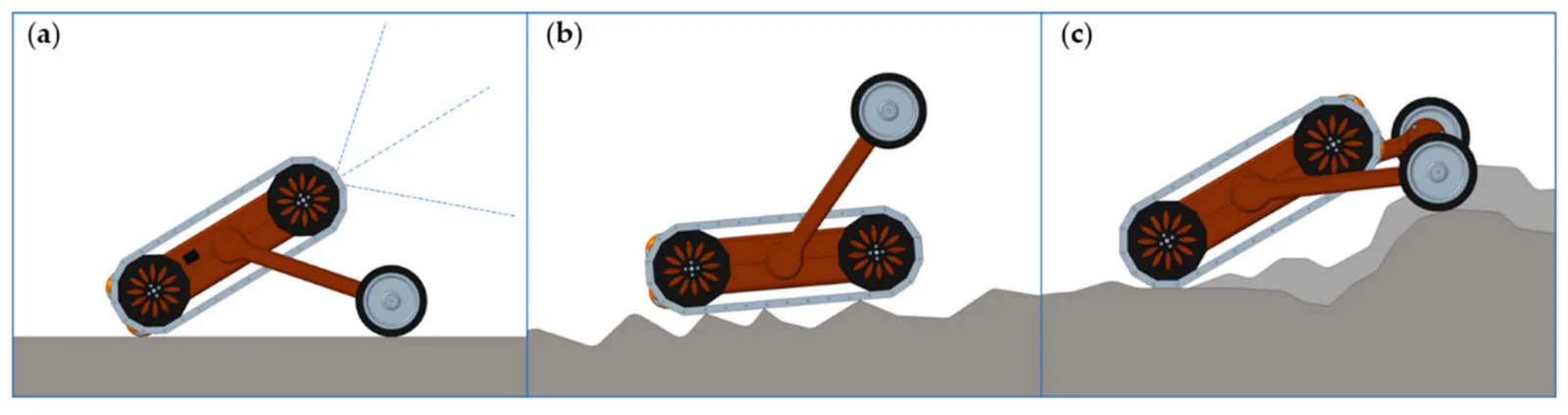
Operation of the Hybrid Model (**a**) Wheeled locomotion on flat and compact surfaces; (**b**) tracked locomotion on yielding terrains; (**c**) hybrid leg-wheel-track locomotion to overcome obstacles [16].

Figure 2 shows a robot platform that can switch between different drive modes depending on the terrain conditions. The mode switching is enabled by an additional built-in motor.

In another design concept, the platform incorporates both tracked and wheeled propulsion, and the selected mode depends on the direction of motion. The two locomotion modes are arranged along two orthogonal axes, ensuring that they can operate independently. In such a configuration, the robot can move longitudinally using tracks and laterally using wheels [17].

For wheeled mobile platforms, several types can be distinguished based on wheel arrangement and steering capability, including Ackermann-type and differential-drive robot platforms, as well as their enhanced and hybrid variants.

A differential-drive robot typically employs two independently actuated drive wheels. Turning is achieved by controlling the speed difference between the two wheels (Equation (1) [18]). These platforms may be complemented by a passive caster wheel at the front or rear, which does not contribute to propulsion [19], as follows:

$$\dot{\theta }=r\left(\frac{\omega_{R}-\omega_{L}}{d}\right)$$

(1) $$\dot{x}=v\cos\theta$$

$$\dot{y}=v\sin\theta$$

where

r: wheel radius. 

$\omega_{R}:\;angular\;velocity\;right\;wheel$. 

$\omega_{L}:\;angular\;velocity\;left\;wheel$. 

d: distance between wheels. 

v: linear velocity. 

**Figure 3:**
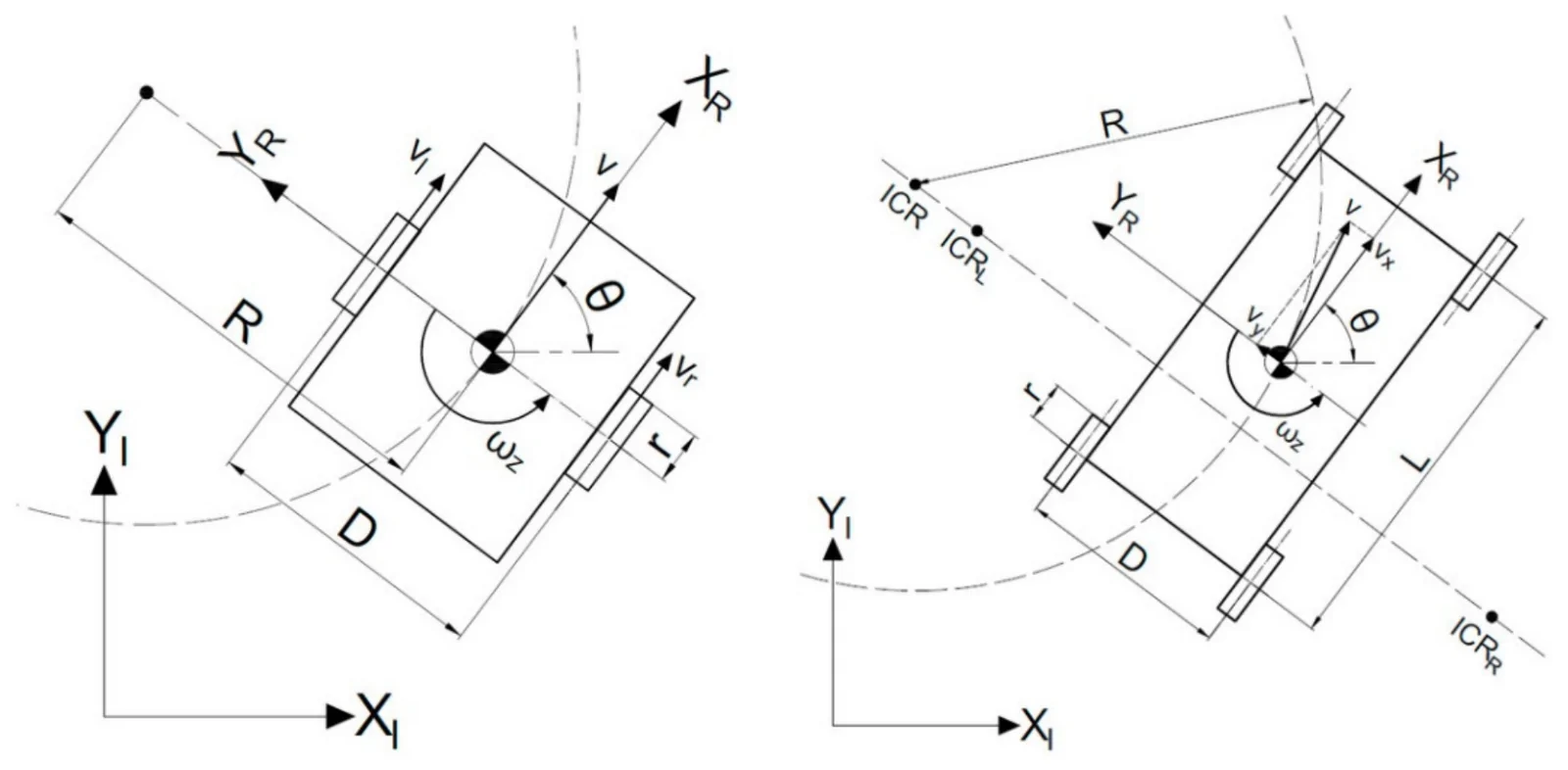
Comparison of Two-Wheeled (**left**) and Four-Wheeled (**right**) Differential Drive [21]. Dashed lines indicate the turning radius (R) and the circular trajectory about the instantaneous center of rotation.

Several enhanced variants of differential-drive robots have also been proposed, in which the platform is equipped not with two but with four or more wheels (Figure 3). In these configurations, all wheels are driven, and turning is likewise achieved by controlling differences in the rotational speeds of the wheels [20].

The operating principle of an Ackermann-type platform is similar to that of passenger cars: the rear wheels provide propulsion, while the front wheels are used for steering and are not driven. The key idea is that, during turning, the front wheels steer at different angles, so that each wheel rolls around a common instantaneous center of rotation. This geometry enables stable cornering with minimal lateral slip [22].

**Figure 4:**
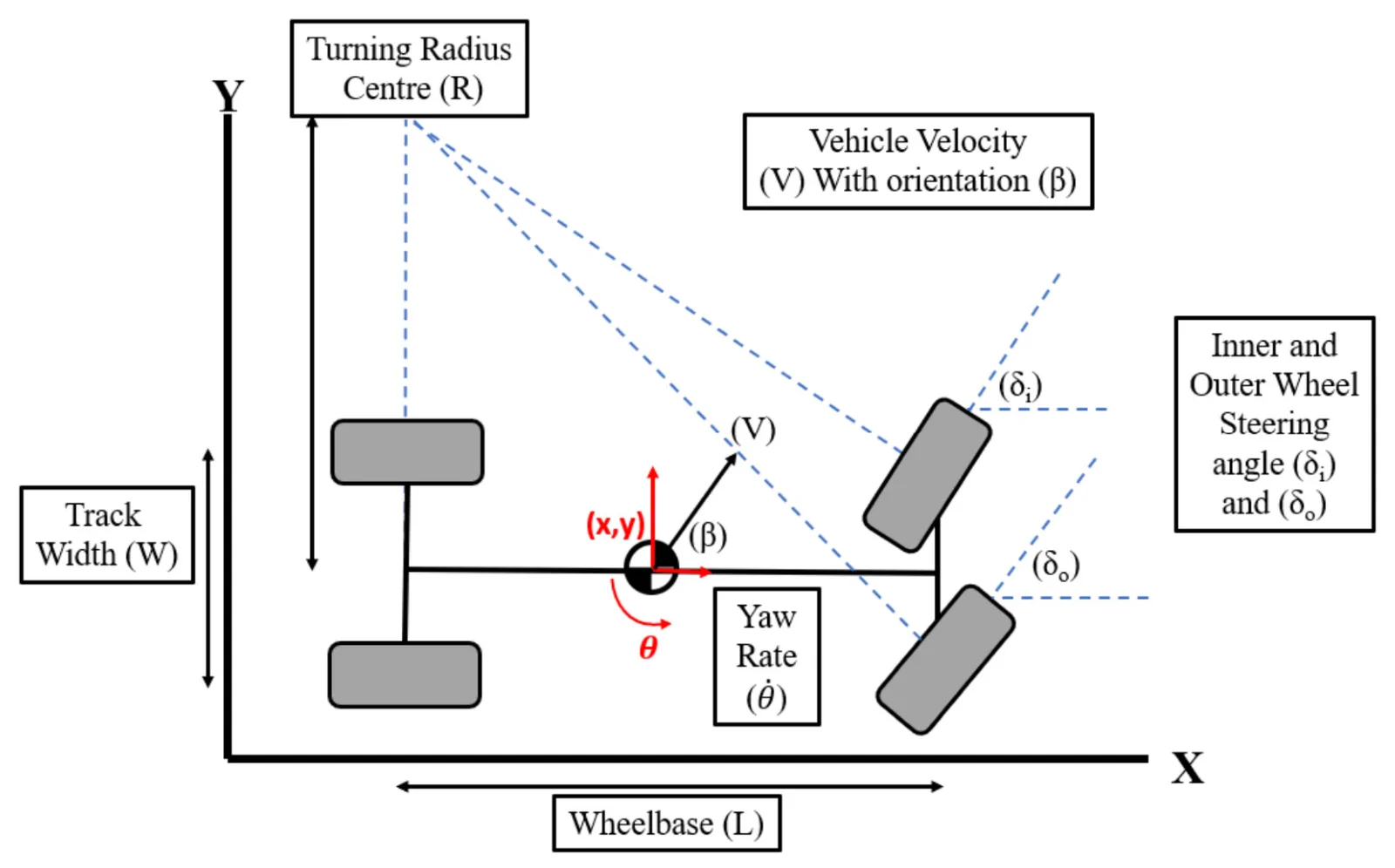
Kinematic Model of Ackermann Steering [24].

The geometric relationships between the different steering angles in the kinematic model of the Ackermann-steering platform are illustrated in Figure 4 and Equation (2) [23], as follows:

$$\omega =\frac{v}{r}i$$

$$R=\frac{l}{\tan\alpha }$$

(2) $$\omega_{L}=\omega \left(1+\frac{d}{2R}\right)\;\omega_{R}=\omega \left(1-\frac{d}{2R}\right)$$

where

v: linear velocity. 

r:wheel radius. 

i: total gear ratio between the motor and the wheel. 

l: wheel base. 

α: steering angle. 

d: wheel spacing. 

$\omega_{R}:\;angular\;velocity\;right\;wheel$. 

$\omega_{L}:\;angular\;velocity\;left\;wheel$. 

In motion-planning problems, the Ackermann steering model is often simplified to the so-called bicycle model, in which the two front wheels are represented by a single steered wheel and the two rear wheels by a single driven wheel. This simplification is used only for planning purposes; the physical robot remains an Ackermann-steered vehicle [25].

**Figure 5:**
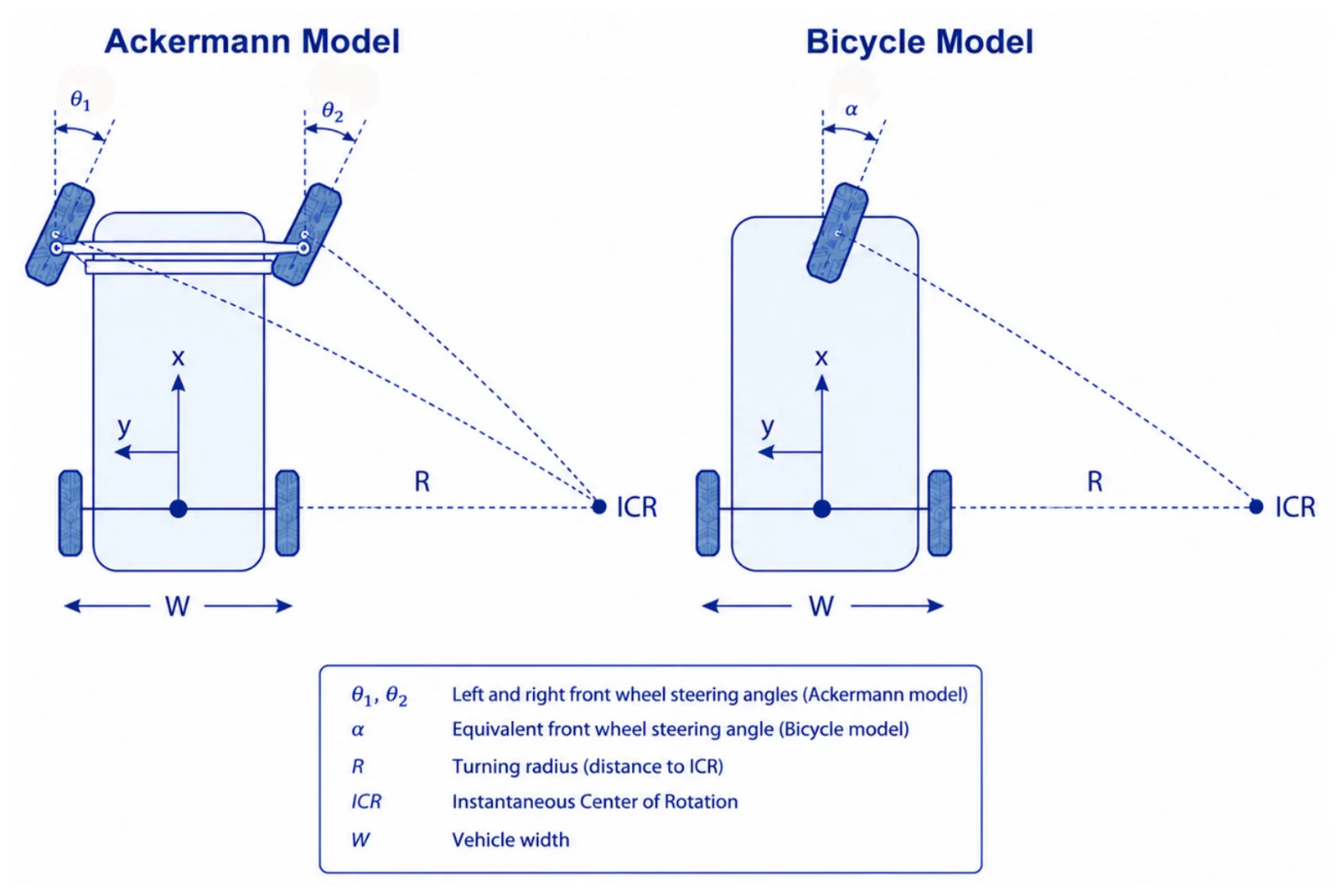
Ackermann Steering vs. Bicycle Model. Adapted from [25].

The relationship between the two models is illustrated in Figure 5.

**Figure 6:**
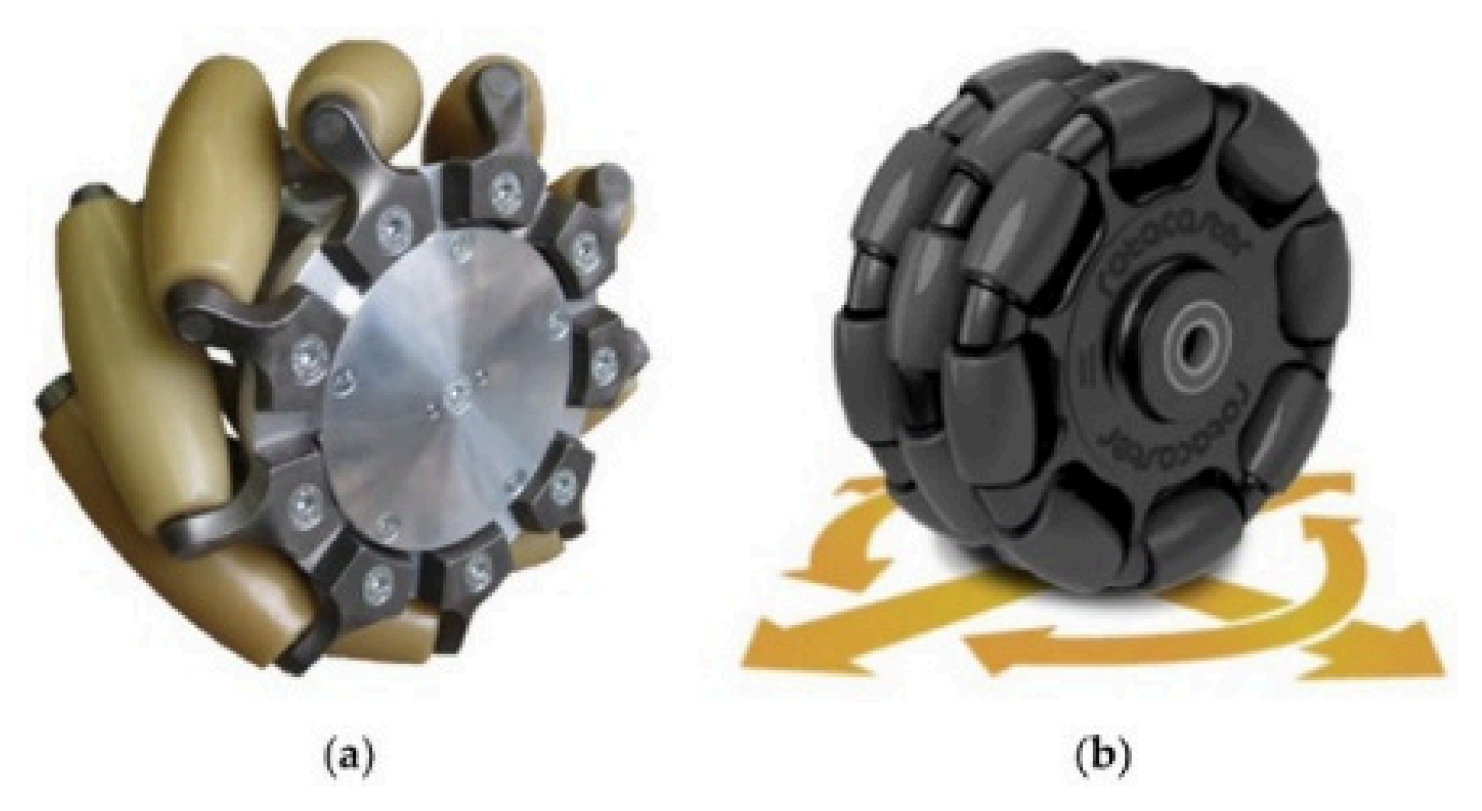
(**a**) Mecanum wheel, (**b**) omni wheel [26]. The yellow arrow in (**b**) indicates the wheel’s free rolling direction, which enables lateral (sideways) motion in addition to standard rotation.

A special variant of differential-drive platforms is represented by robots equipped with Mecanum or omni wheels (Figure 6). These wheels are designed to enable omnidirectional motion, allowing the robot to translate in any direction without needing to rotate its chassis. The underlying principle is that rollers mounted on the wheel circumference permit lateral slip in a controlled manner, which makes sideways motion possible. In omni wheels, the rollers are oriented perpendicular to the wheel plane, whereas in Mecanum wheels they are typically mounted at a 45° angle [26].

In the case of these platforms, the change in travel direction is not achieved through conventional steering, but by differentially controlling the rotational speeds of the individual wheels.

**Figure 7:**
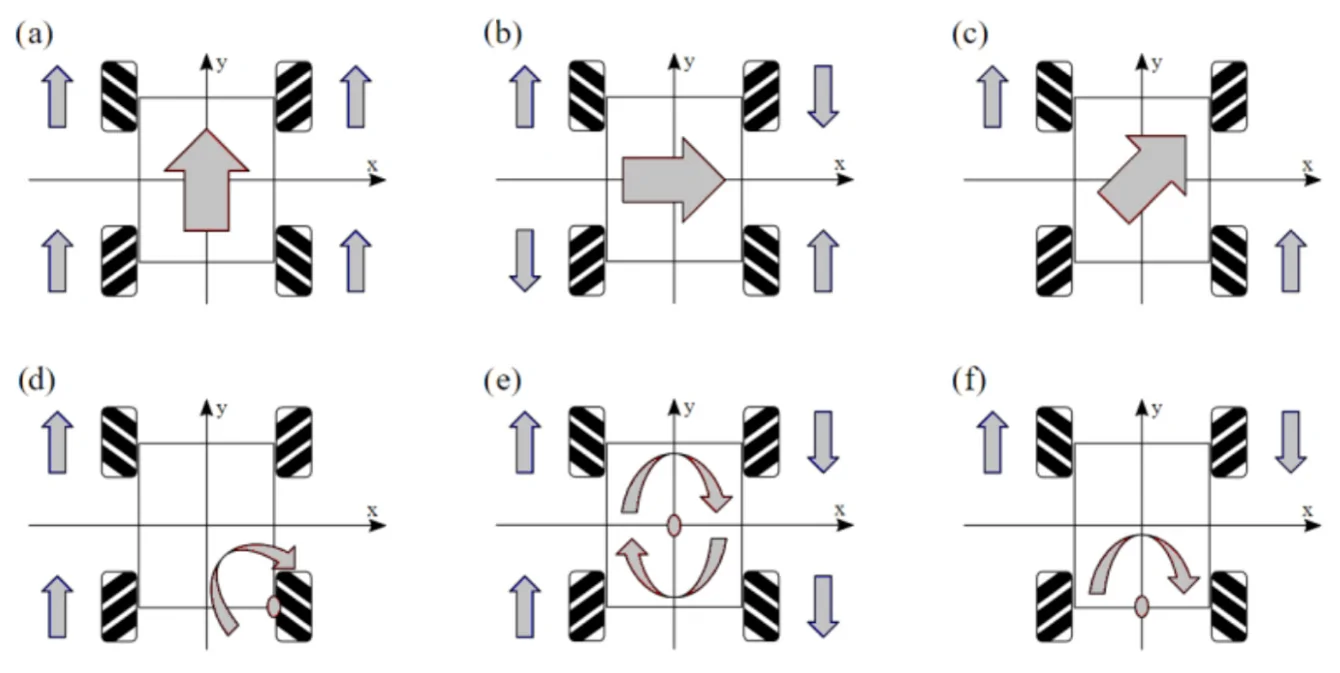
Movement of a Mecanum Wheel-Based Platform [27]. (**a**) forward motion; (**b**) lateral (sideways) motion; (**c**) diagonal motion; (**d**) rotation in place; (**e**) spin motion; (**f**) combined curved motion. Straight arrows indicate individual wheel rotation direction; curved arrows indicate the resulting motion path of the platform.

Figure 7 illustrates the directions and combinations in which each wheel must be driven to achieve the various motion directions.

**Figure 8:**
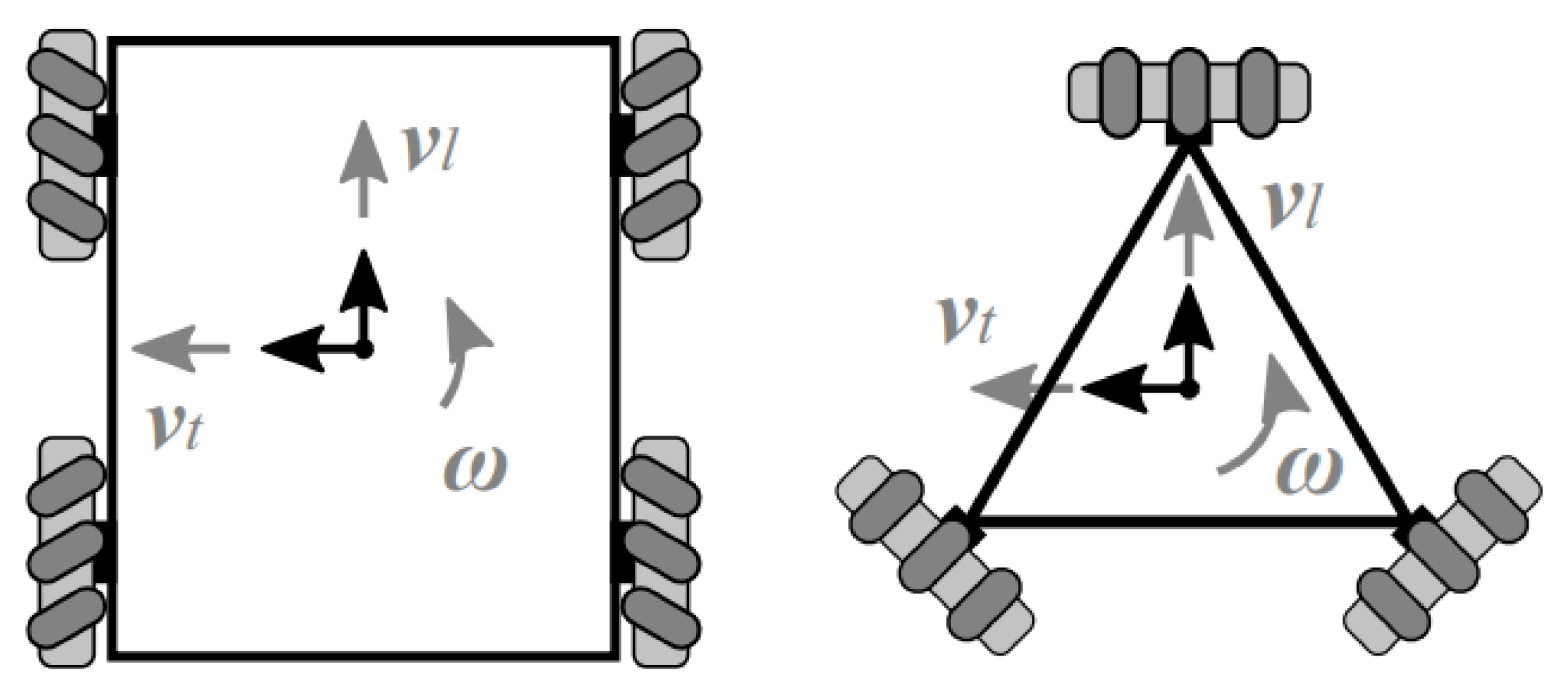
Three- and Four-Wheeled Platforms [28].

Mecanum- and Omni wheel-based robot platforms can be designed with different numbers of wheels, most commonly three or four, but multi-wheel configurations also exist. The drive and control methods always depend on the number of wheels and their geometric arrangement (Figure 8).

Each of these platform types has been applied in practice with distinct trade-offs. Differential-drive robots have been used for field crop/soil monitoring [29] and yield estimation [30], offering simple, low-cost control at the expense of higher lateral tire stress during turning. Ackermann-steered robots have been applied for autonomous cotton-field navigation, providing stable, high-speed tracking but requiring a larger turning radius [31]. Mecanum-wheeled platforms have been used for greenhouse path planning, enabling in-place rotation and lateral motion in narrow rows, though they perform poorly on rough, unstructured outdoor terrain [32].

### 3.3. Platform Types and Typical Tasks

The use of agricultural robots offers numerous advantages and is therefore becoming increasingly widespread as demand grows for more efficient farming practices. Lightweight robot fleets can reduce soil compaction caused by heavy tractors, thereby potentially extending the service life and productivity of arable land. They also enable more precise application of pesticides and herbicides. Precision agriculture is fundamentally based on data-driven decision-making, the use of diverse sensors, and automated data processing; together, these support more efficient and sustainable use of resources [29,33].

Robots are used for in-field soil and crop monitoring. A soil probe mounted on the robot can automatically adjust to the terrain slope, enabling more accurate soil measurements. A camera mounted on a robotic arm can recognize different crop growth stages and visually assess plant health from captured images. The system can also be used to determine plants’ physical characteristics [29].

A robot equipped with a camera and a spraying system can apply chemicals in a targeted manner only to the desired plants, significantly reducing unnecessary chemical use. The onboard camera captures images of crops in real time, which are processed by a trained deep-learning, YOLO-based model to identify plants and estimate their precise locations. The coordinates determined by the vision model are then processed by the spray-control system, which activates the valve corresponding to the detected position. If each nozzle can be controlled independently, the system treats only the targeted plants. Compared to conventional broadcast spraying, this approach can reduce pesticide use by up to 60% [34].

Three-dimensional LiDAR can also be used for detection. LiDAR has been applied to sense spatial information about fruit trees in the robot’s surroundings and to delineate the area to be sprayed. The system combines PWM technology with a precision variable spraying system (PVS), improving upon conventional continuous spraying by enabling targeted application adapted to the detected surface. As a result, pesticide use is reduced, as are spray drift into the air and losses to the ground [35].

**Figure 9:**
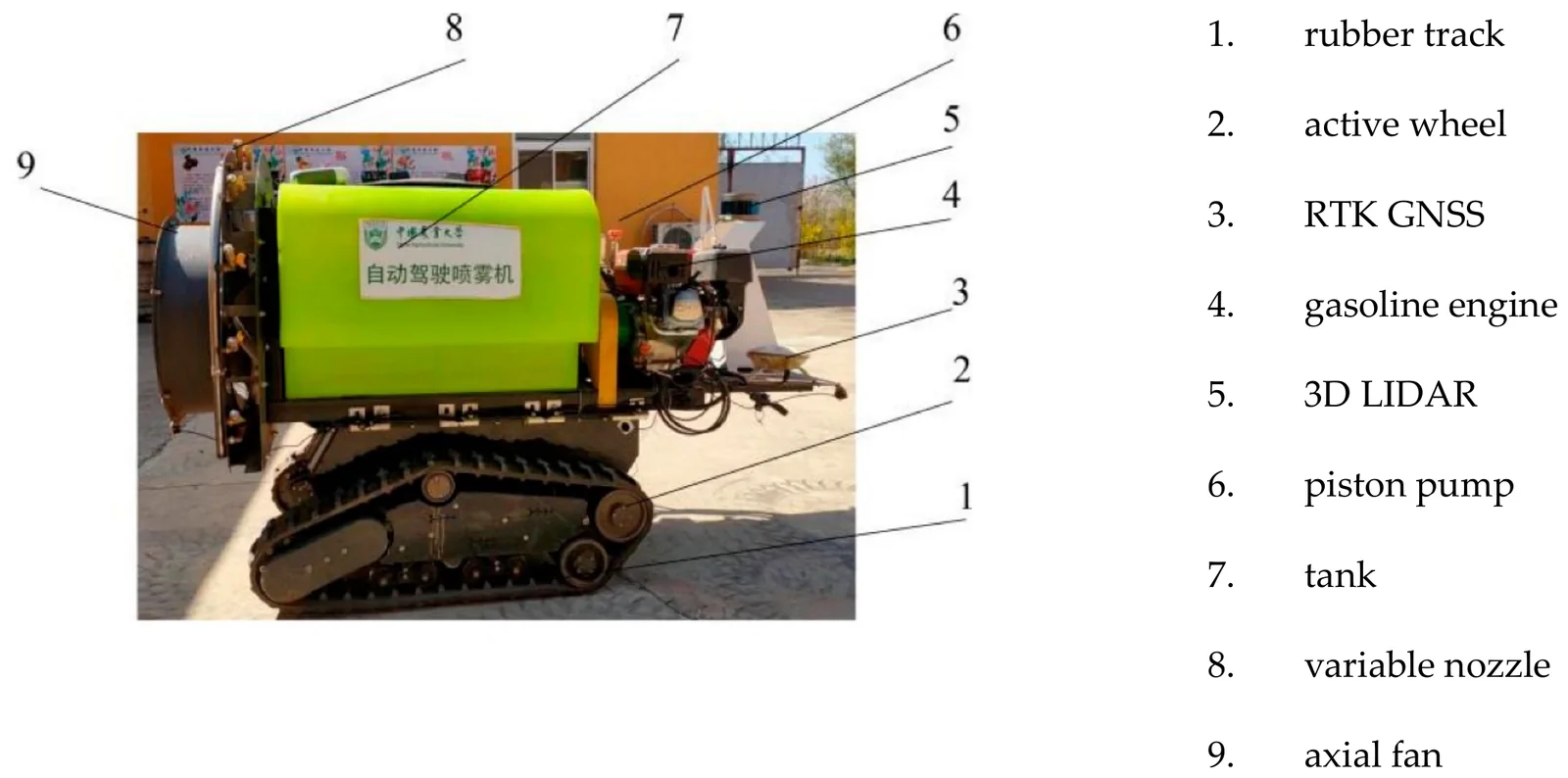
Spraying Robot [35]. The Chinese text on the housing reads "Autonomous Driving Sprayer".

The spraying robot utilizing this technology is shown in Figure 9.

An autonomous robot equipped with machine vision can be used for yield estimation, which has been successfully demonstrated, for example, in tomato and peanut plantations. The robot performs plot-level data collection and then uses high-resolution cameras and depth sensors to capture plant structure, fruit count, and plant height, thereby enabling more accurate yield prediction. A key advantage of non-destructive phenotyping is that repeated measurements can be performed on the same plots without damaging the plants, allowing continuous monitoring throughout the growing season. Compared with conventional manual sampling, an autonomous robot-based approach can substantially reduce labor requirements while preserving crop integrity [30,36].

## 4. Navigation Systems and Algorithms

The transition from manual operation to full autonomy in agriculture hinges on the robot’s ability to perceive its surroundings and execute precise movements. A robust navigation system must bridge the gap between high-level mission goals—such as covering an entire vineyard—and low-level actuator commands. This process is inherently hierarchical: it begins with understanding the environment through sensor data, proceeds to calculating a collision-free path, and concludes with the real-time control of the vehicle’s motors. The following sections detail the modular architecture of these systems, the distinction between global and local planning, and the mathematical frameworks that ensure accuracy even under challenging field conditions.

### 4.1. Modules of a Robot Navigation System

When designing a robot navigation system, it is important to distinguish between trajectory planning and motion planning [37].

The goal of trajectory planning is to determine the route the robot should take from one point to another. These points may be given as GPS coordinates or as positions defined in a local map. Trajectory planning must account for the traversable area, the robot’s current pose and target pose, the robot’s orientation, the desired arrival orientation, and the robot’s physical properties. Important properties include the robot’s size and steering capability, from which the turning radius can be derived, indicating how much space the robot requires to execute a turn. Trajectory planning can be formulated as an optimization problem, where typical objectives include minimizing time, energy consumption, and overall motion [38,39,40].

In motion planning or trajectory tracking, the objective is to determine how the robot can follow the previously planned trajectory. This includes computing the linear (driving) and angular (steering) velocities. To determine these control commands, the robot’s kinematic parameters must be considered, including acceleration limits as well as maximum linear and angular speeds [41,42].

**Figure 10:**
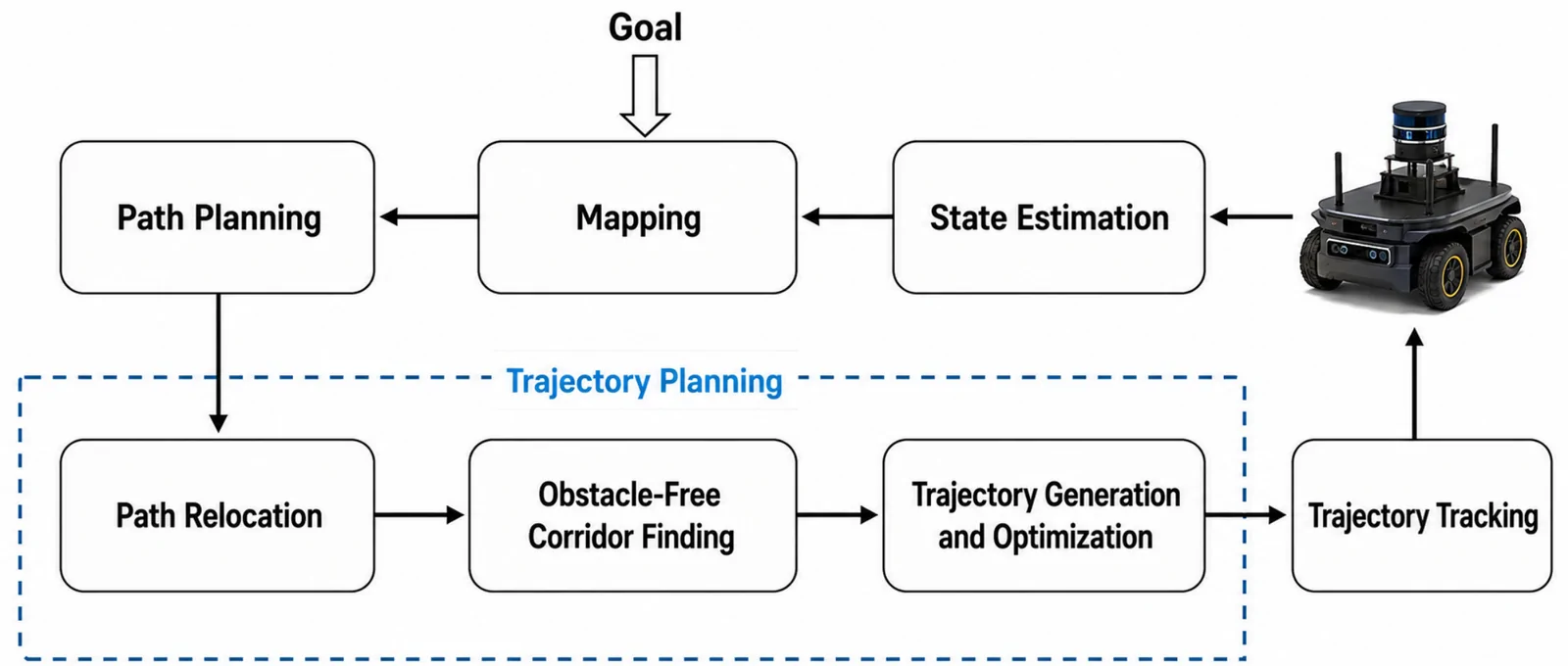
Trajectory Planning and Tracking [44].

Trajectory planning and motion planning are closely coupled processes that influence each other (Figure 10). Results from motion planning can provide feedback to refine the trajectory, while changes to the trajectory may, in turn, necessitate additional motion-planning steps [43].

### 4.2. Global Trajectory Planning

Trajectory planning is typically performed at two levels: global and local. Global trajectory planning is a map-based route-planning process that determines a complete path from the start point to the goal point according to a specified objective, such as travel time, path geometry, or energy consumption. Based on the information available in a map, this process accounts for traversable regions and known obstacles in advance [45].

Global planning can be carried out on a geographic map; however, such maps are often unavailable for agricultural areas.

**Figure 11:**
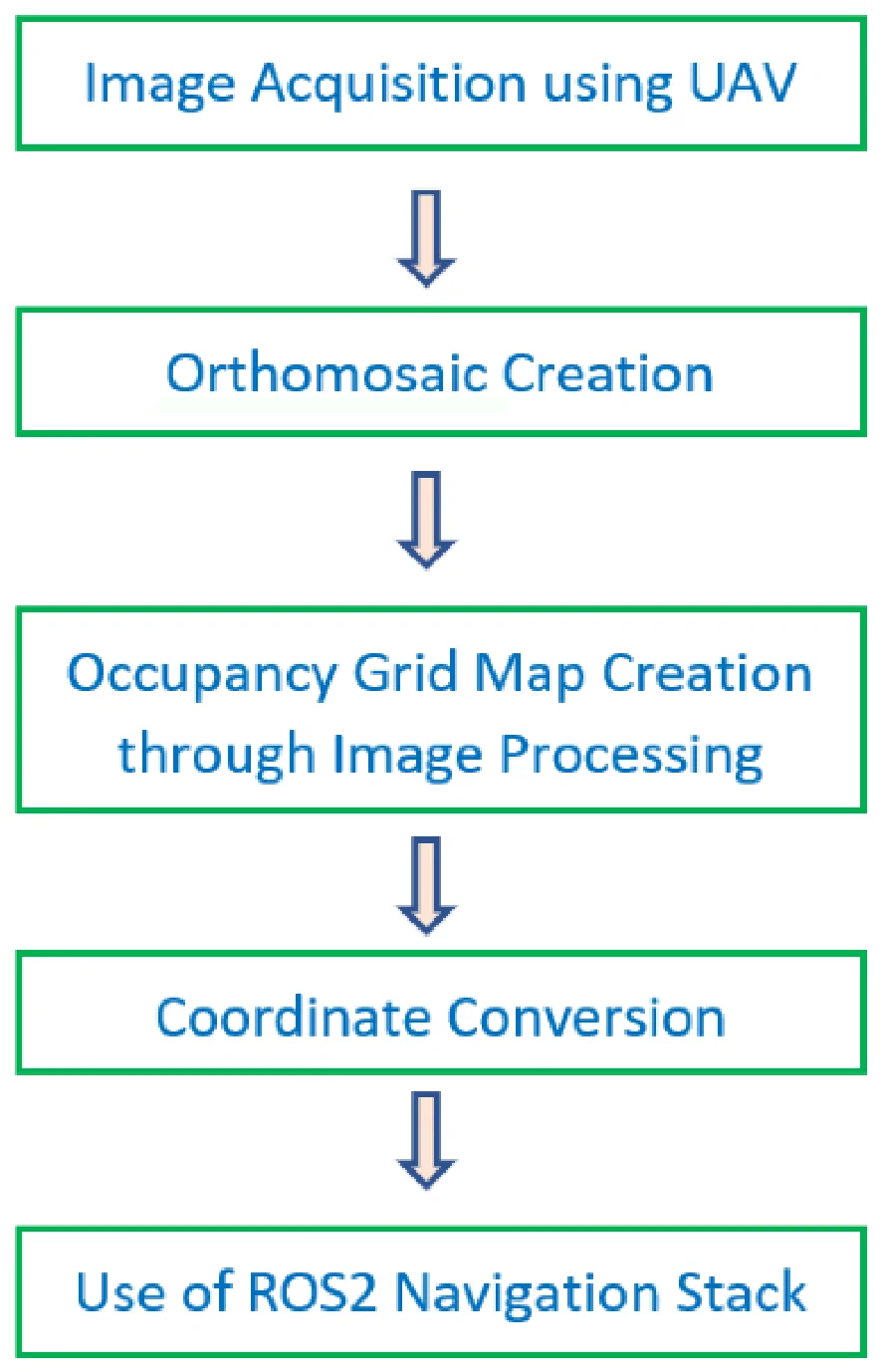
Map Generation [46].

Therefore, the required map can also be generated from local UAV imagery. The drone captures images of the agricultural area along a pre-planned flight path, from which an orthomosaic image can be produced by stitching overlapping images and correcting distortions. Using image processing, an occupancy grid map can then be constructed to distinguish free traversable space from obstacles (Figure 11). By registering this grid map in the UTM coordinate system, a map can be obtained that is suitable for autonomous navigation of agricultural robots [46].

The required map can be generated using environmental mapping algorithms implemented on the mobile robot, which construct the map while the robot is moving. Alternatively, the area can be mapped using drones.

Several algorithms are available for environmental exploration. One of the earliest and still widely used methods is Frontier-Based Exploration, which also provides the basis for several more recent algorithms. Its core principle is that the robot continuously identifies frontier points located at the boundary between mapped and unknown areas. It then determines whether a frontier point represents traversable space or an obstacle and moves along the traversable route toward the next frontier target. The robot updates the map after every new measurement. This process is repeated until no unexplored area remains.

When selecting the next target, more recent frontier-based approaches consider not only distance but also expected information gain. Rather than exhaustively examining every point, they use a path planner based on the information already available, for example, the A* algorithm. This approach results in a shorter exploration time than the classical frontier-based algorithm [47].

Several enhanced variants of frontier-based algorithms have been developed in which the conventional approach is combined with other methods to improve processing time [48].

Recent research has also applied deep-learning algorithms to environmental exploration. In these approaches, the robot does not select the next target on the basis of predefined heuristics; instead, through interaction with the environment, it learns which decisions lead to the most efficient exploration. Deep-learning approaches can also adapt to complex, dynamic environments [49,50].

During exploration, however, selecting the next target alone is insufficient. The robot must also continuously construct an accurate map of its environment while determining its own position. This task is performed by Simultaneous Localization and Mapping (SLAM) algorithms [51].

In agricultural environments, particularly where GNSS availability is limited, such as in greenhouses, SLAM plays a key role in autonomous navigation by integrating LiDAR, camera, and odometry data [51].

To determine the robot’s global position, an RTK-enabled GNSS (GPS) system can be used, while orientation can be obtained using an IMU (Inertial Measurement Unit) sensor [52].

An IMU is an electronic sensing unit that combines accelerometers and gyroscopes to measure the robot’s three-dimensional orientation, providing yaw, pitch, and roll estimates [53].

**Figure 12:**
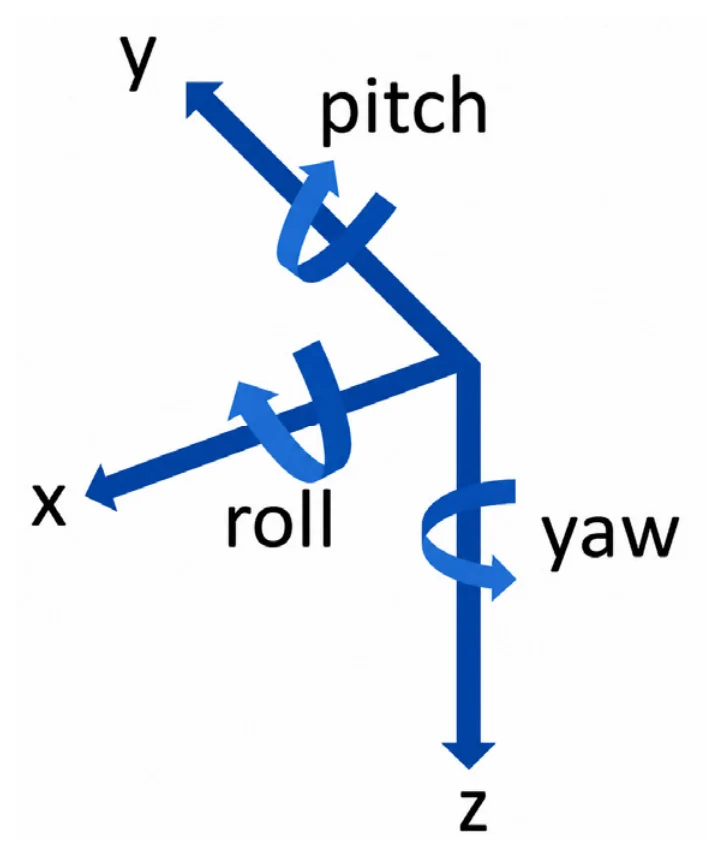
Representation of Euler Angles: Yaw, Pitch, and Roll [54].

Figure 12 illustrates how yaw (rotation around the vertical axis), pitch (rotation around the lateral axis), and roll (rotation around the longitudinal axis) result in changes in spatial orientation.

A well-known phenomenon affecting IMUs is so-called drift. This means that the orientation values provided by the sensor gradually shift over time, even when the robot is stationary. During motion, this drift can accumulate into a growing measurement error, degrading localization and navigation accuracy. Drift can be mitigated in software, for example by setting an initial offset and applying velocity-based resets during operation [55].

Estimating sensor zero-offset errors and using them for initial calibration can improve short-term measurement accuracy; however, these measures alone cannot eliminate drift that accumulates over the long term. Bias is a systematic sensor measurement error that indicates the extent to which the mean of the measured values deviates from the reference value. Therefore, continuous bias estimation and bias compensation are applied in practice to reduce drift [56].

To further improve long-term accuracy, IMU data are generally fused with RTK-assisted GNSS or data from other sensors using sensor-fusion methods to compensate for drift [57].

A basic IMU measures linear acceleration and angular velocity along three axes (x, y, z), from which orientation can be estimated. In mobile robotics, 6-degree-of-freedom (6-DOF) and 9-degree-of-freedom (9-DOF) IMUs are widely used. A 6-DOF IMU includes a tri-axial accelerometer and a tri-axial gyroscope, whereas a 9-DOF IMU additionally incorporates a tri-axial magnetometer. Measurements from these different sensors are combined using sensor-fusion algorithms, which reduce noise and the impact of drift, thereby improving orientation estimation. With an IMU that includes a magnetometer, orientation can be determined not only locally but also in a global reference frame [58].

**Figure 13:**
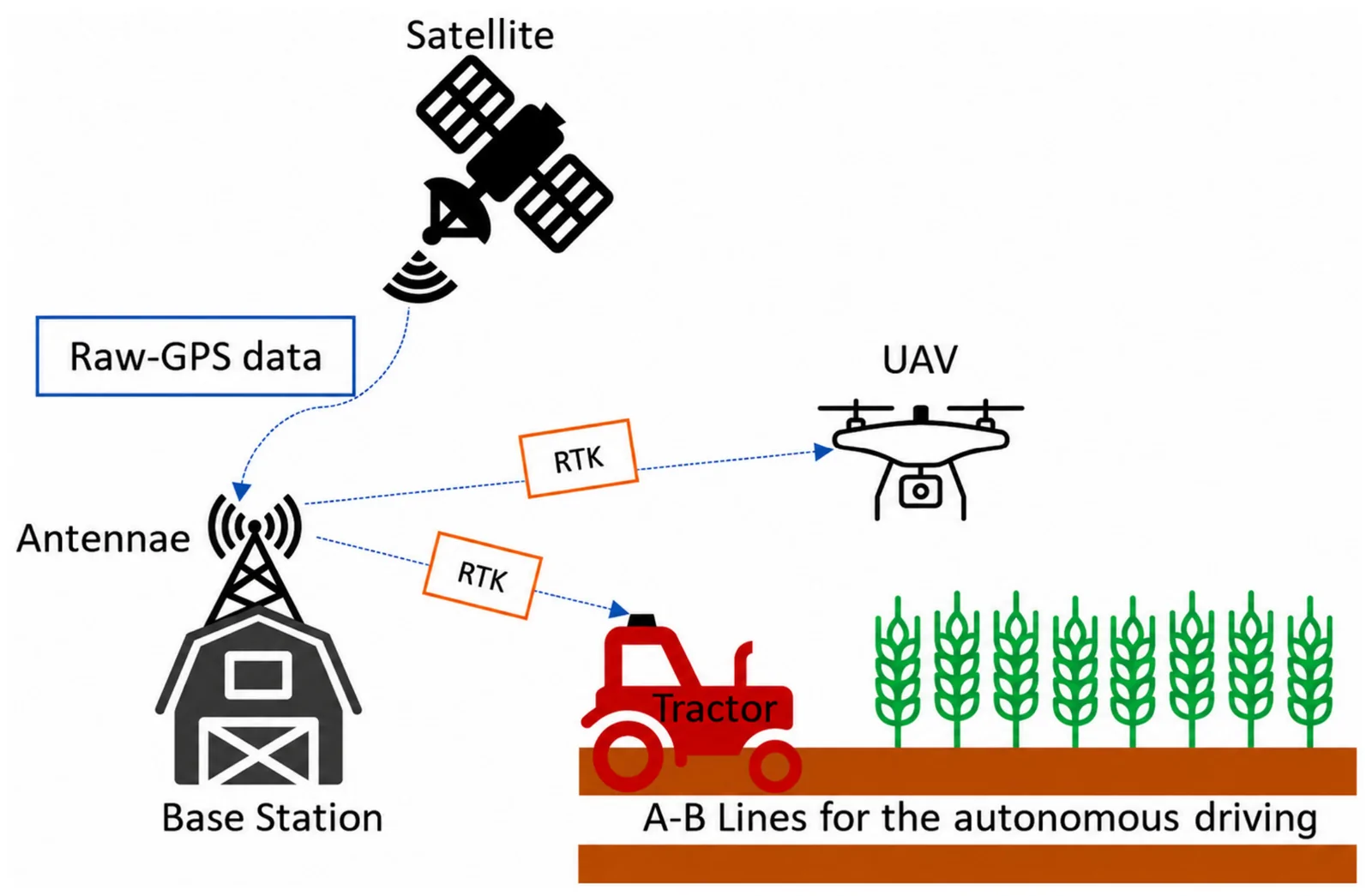
Operation of RTK (Real-Time Kinematic) [59].

The purpose of Real-Time Kinematic (RTK) technology is to improve the accuracy of satellite-based positioning. This is particularly important in domains such as agriculture, where high precision is essential for path tracking. An RTK system consists of two main components: a base station and a rover (Figure 13). The base station is a GNSS receiver installed at a fixed location, while the rover is the GNSS module mounted on the moving platform, in this case, the robot. The base station continuously receives satellite signals and transmits correction information to the rover in RTCM (Radio Technical Commission for Maritime Services) format, for example via an NTRIP (Network Transport of RTCM via Internet Protocol) server. Using these corrections, the rover refines its own GNSS measurements, enabling centimeter-level positioning accuracy under suitable conditions [59].

### 4.3. Local Trajectory Planning

Local trajectory planning is required in dynamic or unknown environments. The algorithm modifies the robot’s path in real time based on sensor data to safely avoid obstacles that may appear unexpectedly [60].

**Figure 14:**
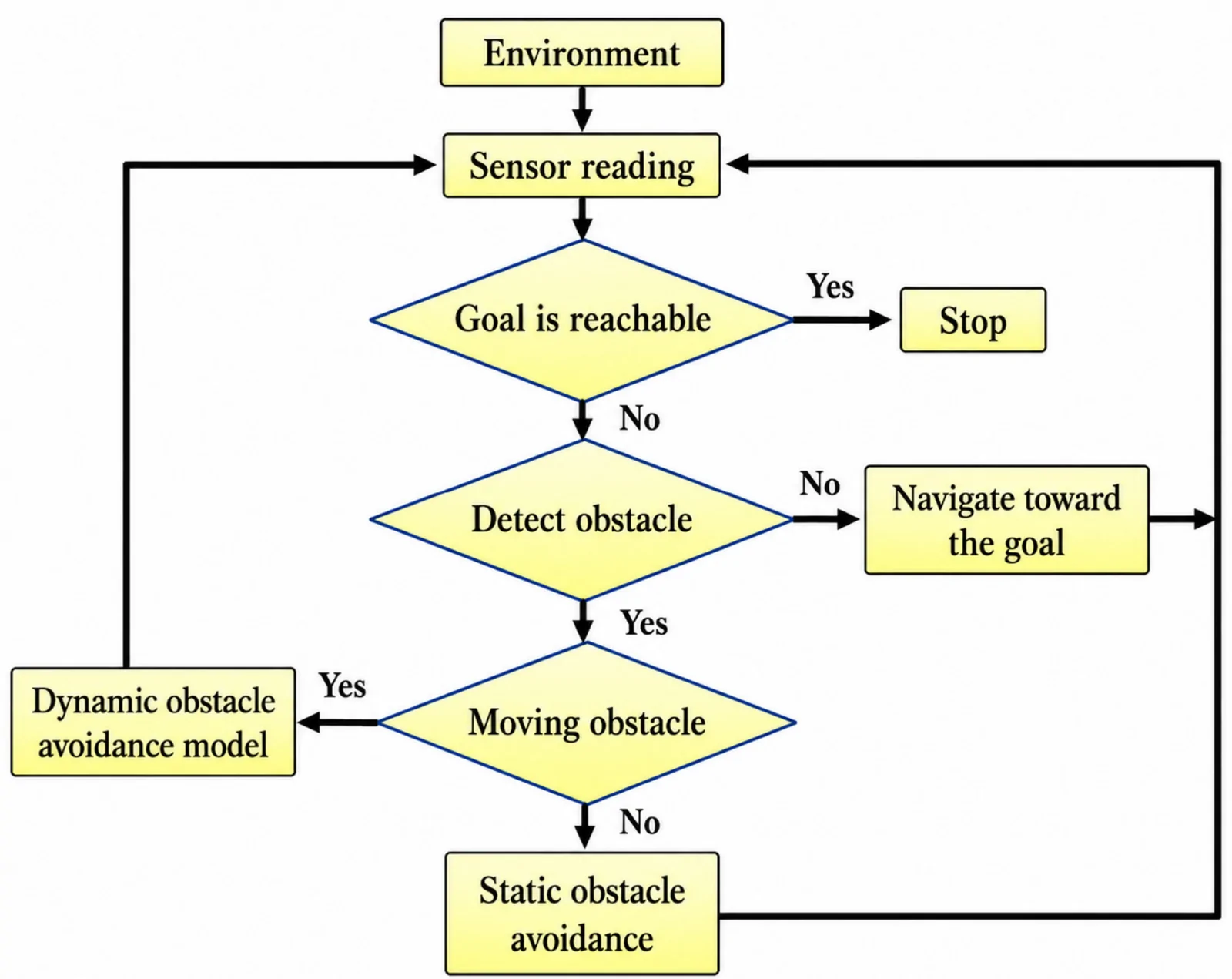
Obstacle Avoidance [60].

The flowchart of the dynamic obstacle avoidance algorithm presented by Katona is illustrated in Figure 14.

The goal of local planning is to enable the robot, when necessary, to modify the globally planned route while still maintaining the overall global direction toward the target.

In agriculture, local trajectory planning is especially important because vegetation conditions change continuously, so robots cannot rely solely on pre-recorded maps. In such dynamic environments, the robot must be able to adapt to its surroundings in real time and respond to environmental changes [61].

Local trajectory planning for agricultural robots relies on the combined use of multiple sensors so that dynamically appearing obstacles can be detected reliably and in real time, even under varying crop density, uneven terrain, and different lighting conditions. Such sensors may include LiDAR, radar, ultrasonic sensors, cameras (stereo or thermal), and an IMU [62].

**Figure 15:**
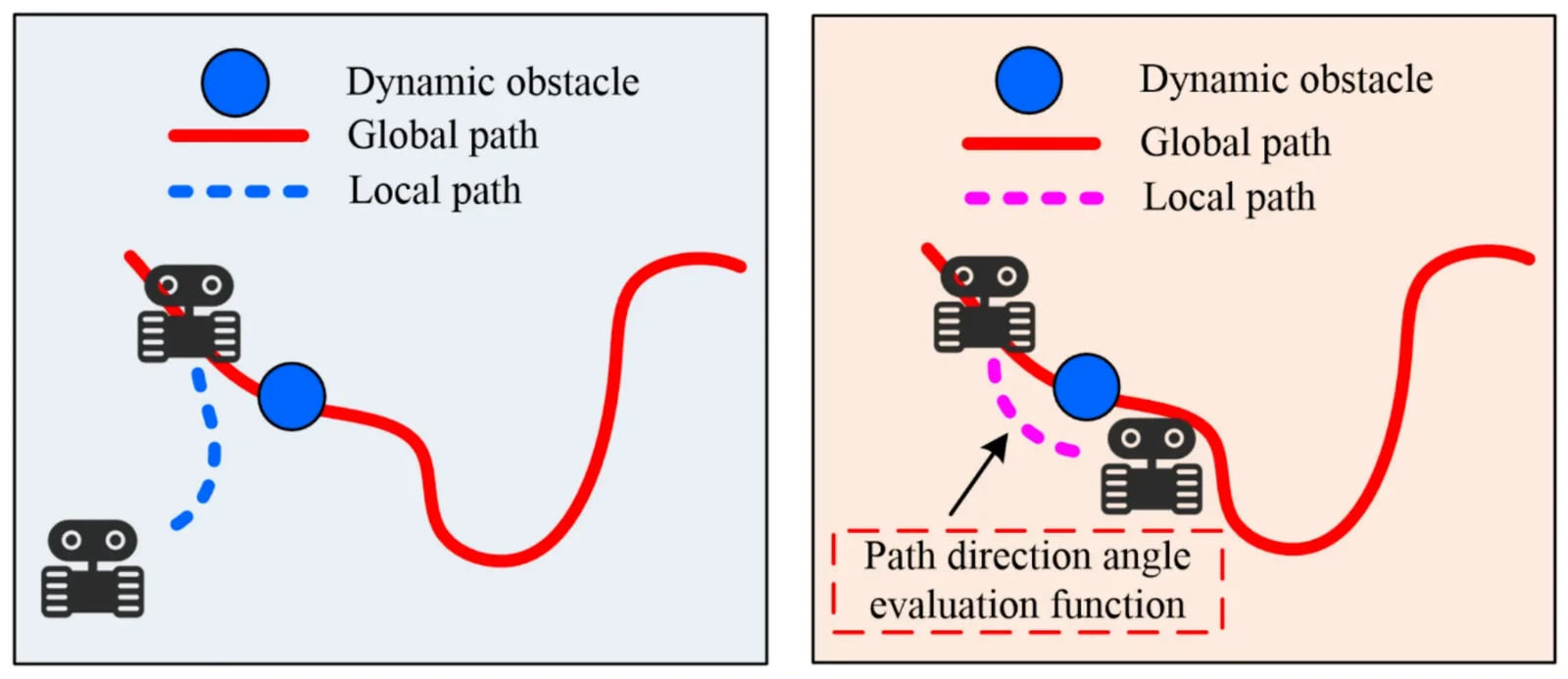
Local and Global Trajectory [63].

Global trajectory planning first determines the robot’s optimal, collision-free route between the start and the goal on a static map. This is followed by local trajectory planning, which runs in real time using the robot’s current sensor data to avoid dynamically appearing obstacles (Figure 15). The two levels continuously influence each other: if local planning diverts the robot from the original route, the system regenerates the global plan so the robot can continue moving toward the target [63].

### 4.4. Trajectory Planning Algorithms

**Figure 16:**
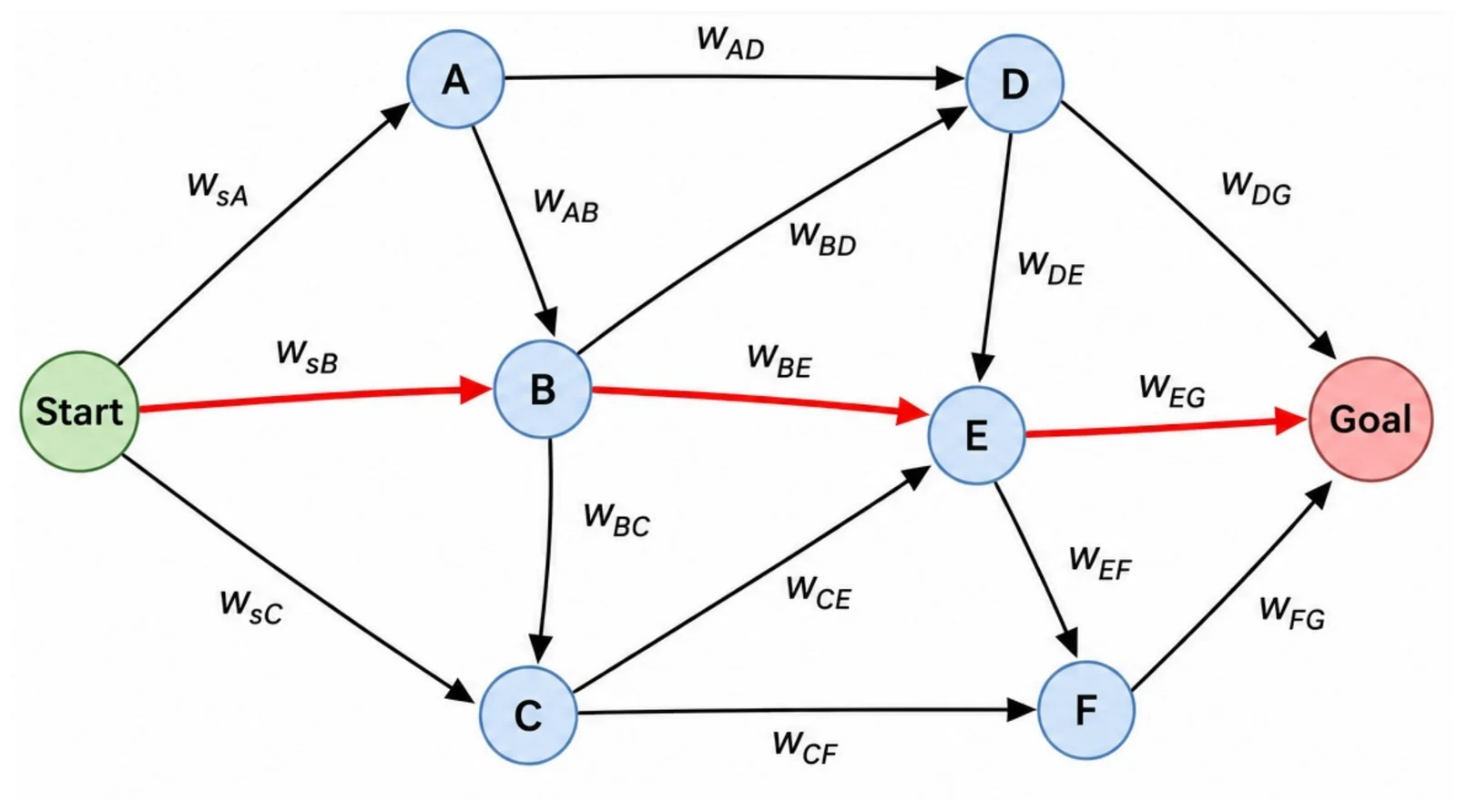
Dijkstra. Adapted from [65]. Nodes A–F represent intermediate states, "Start" and "Goal" denote the initial and target nodes, and $w_{XY}$ denotes the edge weight between nodes X and Y. The red path indicates the shortest route found by the algorithm.

Dijkstra’s algorithm is used to determine the shortest path between a start point and a goal point in a weighted graph (Figure 16). The algorithm, step by step, selects the node with the lowest cost and then updates the distances of neighboring nodes until it has found the shortest path to every node [64]. The method is also applied in robot path planning by converting a map containing obstacles into a graph, allowing the algorithm to compute the robot’s optimal, collision-free route [65].

A* (A-star) is a heuristic search algorithm used primarily for path planning. Its goal is to find the shortest or lowest-cost route between a start point and a goal point on a static map where the locations of obstacles are known. The algorithm processes nodes in order of priority, taking into account both the cost of the path traveled so far and a heuristic estimate of the remaining cost to reach the goal. Optimization is performed using a cost function *f*(*n*) (Equations (3) and (4)) [66]:

(3) f(n)=h(n)+g(n)

where

$g_{n}:\;the\;actual\;cost\;of\;the\;path\;from\;the\;start\;node\;to\;the\;current\;node$. 

$h_{n}:\;the\;estimated\;heuristic\;cost\;from\;the\;current\;node\;to\;the\;goal\;node$. 

The heuristic term is commonly computed using Euclidean distance:

(4) $$h_{n}=\sqrt{{\left(x_{n}-x_{goal}\right)}^{2}+{\left(y_{n}-y_{goal}\right)}^{2}}$$

**Figure 17:**
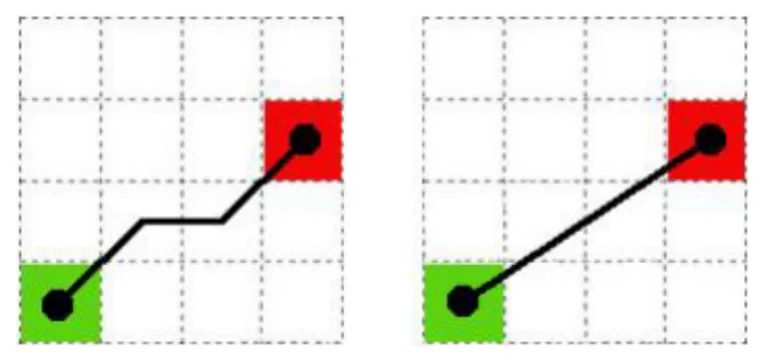
A* Trajectory Planning Error [67]. Green denotes the start point, red denotes the goal point, and the black line indicates the planned path/trajectory.

The A* algorithm is effective for global, static path planning; however, it does not always produce the mathematically shortest route. Because it uses a grid-based approach, the robot can move only in four or eight directions, which results in an angular, piecewise path, so the route is often not optimal (Figure 17) [66,67].

Several improved variants of the classic A* algorithm exist, aiming to smooth paths and make them more natural.

Theta* is an enhanced version of A* that checks whether there is a direct line of sight between two points. If there is, it skips the intermediate points between them. As a result, the generated path becomes more continuous.

Phi* is a further development of Theta*. Phi* assigns direction intervals to each examined point, which define the range of angles along which the planning can proceed. This approach allows the algorithm to account for the robot’s steerability and motion-dynamic constraints during path planning, so the resulting path is not only smoother but also better aligned with the robot’s physical motion [67].

Hybrid A* is an enhanced version of classical A* that considers not only steerability but also reacts in real time to static and dynamic obstacles in the environment. Therefore, it is suitable not only for global but also for local trajectory planning [68].

The Dynamic Window Approach (DWA) is a real-time local trajectory planning algorithm developed for obstacle avoidance in mobile robots. Based on values of linear velocity (v) and angular velocity (ω\omega), the algorithm simulates short predicted trajectories within a dynamic window. It scores these candidate trajectories using an objective function (for example, progress toward the goal or distance from obstacles) and selects the most favorable one for the next step. The robot then moves forward, creates a new dynamic window from the updated current pose, and repeats the process until it reaches the target. This continuous replanning enables real-time adaptation to environmental changes and avoidance of moving obstacles [69,70].

**Figure 18:**
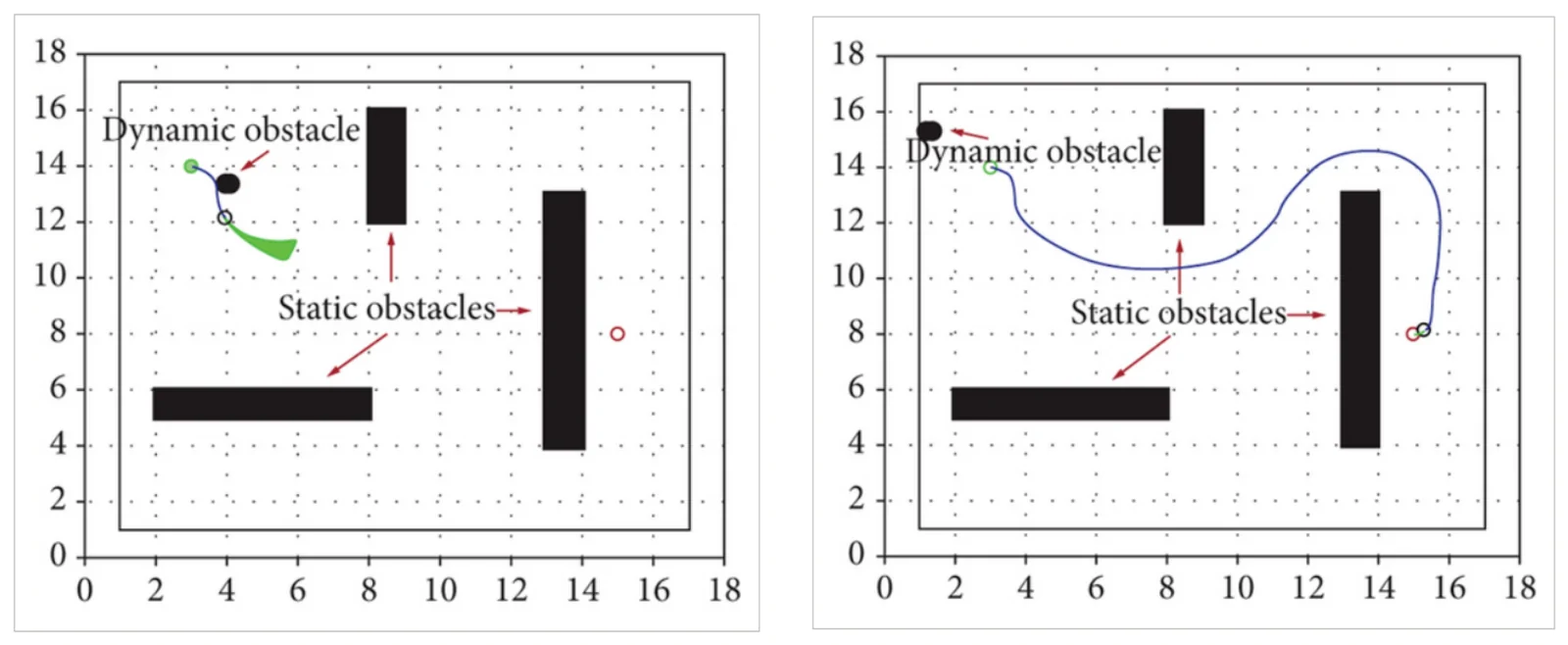
DWA Algorithm [70]. The green shape represents the robot’s current position and orientation; the black dot indicates the dynamic obstacle’s position; black rectangles represent static obstacles; the small red circle marks the goal position; red arrows label the corresponding elements.

Figure 18 illustrates the dynamic path planning of the DWA algorithm. In the first image, the possible paths generated from the current state within the dynamic window are shown in green, from which the algorithm selects the optimal one. The second image shows the path planned after the execution of the DWA.

**Figure 19:**
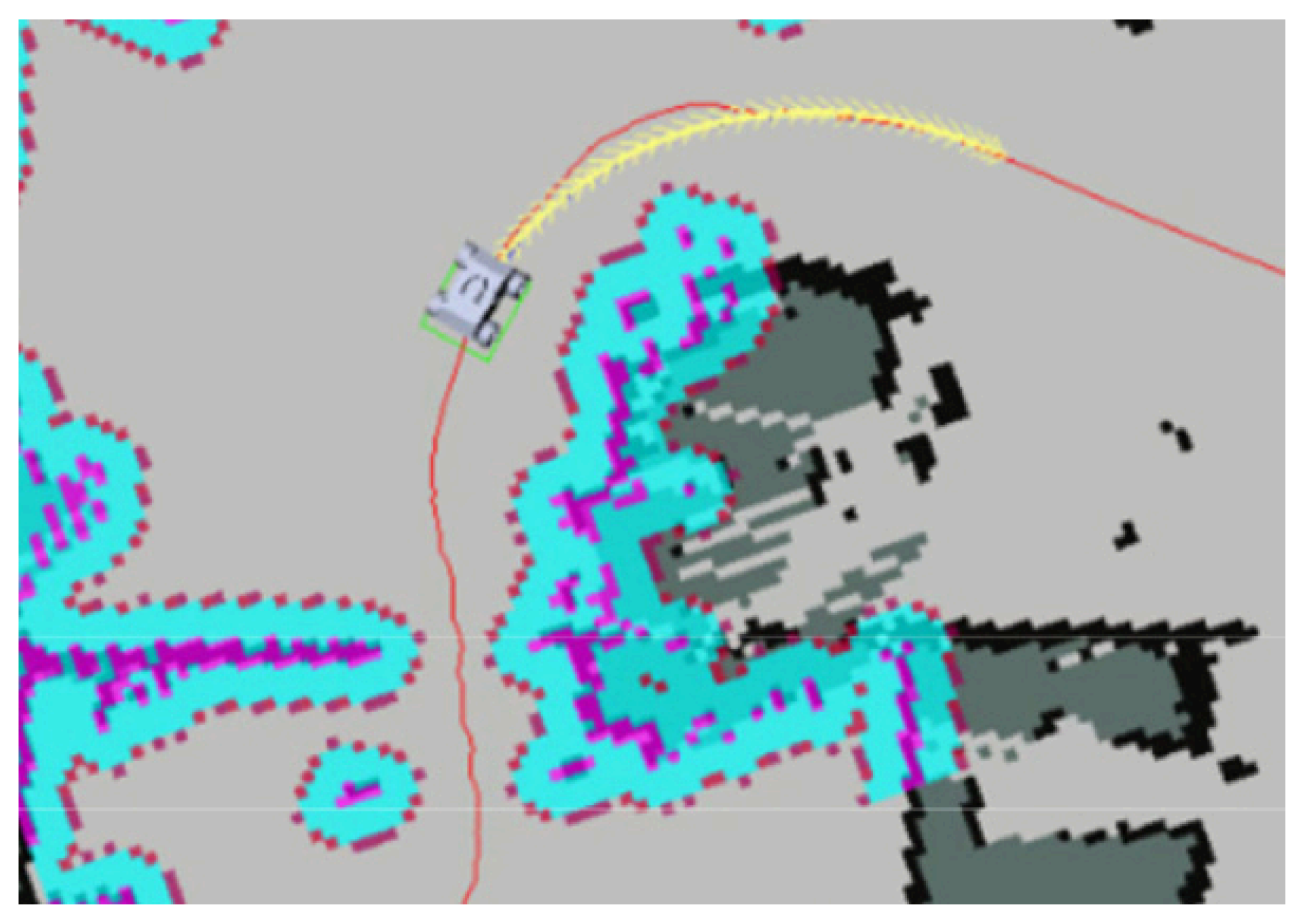
TEB Algorithm. Red line: global path; yellow line: local path planned by the TEB algorithm [71].

The Timed Elastic Band (TEB) algorithm is a real-time, optimization-based local trajectory planner (Figure 19). The algorithm treats the path received from the global planner as a flexible “elastic band” and deforms (stretches) it while taking into account the robot’s physical constraints and obstacle-avoidance constraints [71].

Table 3 presents a comparison of the trajectory planning algorithms described above in terms of planning level, representation, environmental model, and computational complexity.

### 4.5. Motion Control Algorithms

The Proportional–Integral–Derivative (PID) controller is a feedback-based control algorithm that is widely used in industrial control systems and robotics. It can also be used effectively for motion control tasks [76,77].

**Figure 20:**
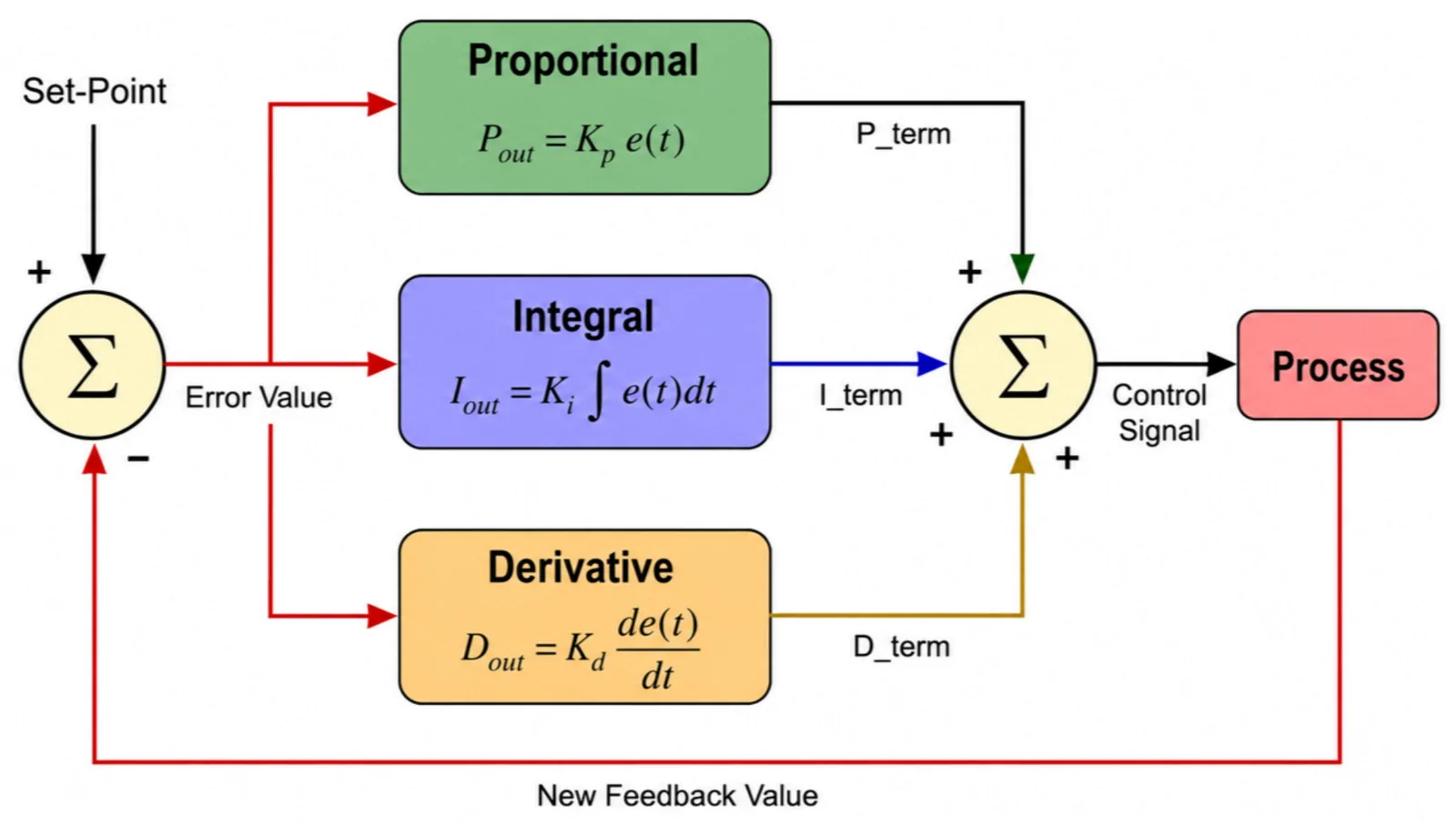
PID Controller [77].

The controller consists of three components (Figure 20):

- The proportional (P) term adjusts the output in proportion to the magnitude of the current error, providing an immediate response to deviations.
- The integral (I) term considers the accumulated sum of past errors, thereby compensating for persistent, steady-state errors.
- The derivative (D) term measures the rate of change in the error, which helps damp overshoot and increases system stability.

In motion control tasks, coordination among multiple PID controllers often provides overall control. For example, separate PID loops can regulate speed and steering. A dedicated PID system can also be used to coordinate throttle and brake control so that the vehicle slows down in sharp turns while allowing maximum acceleration on straight segments [77,78].

**Figure 21:**
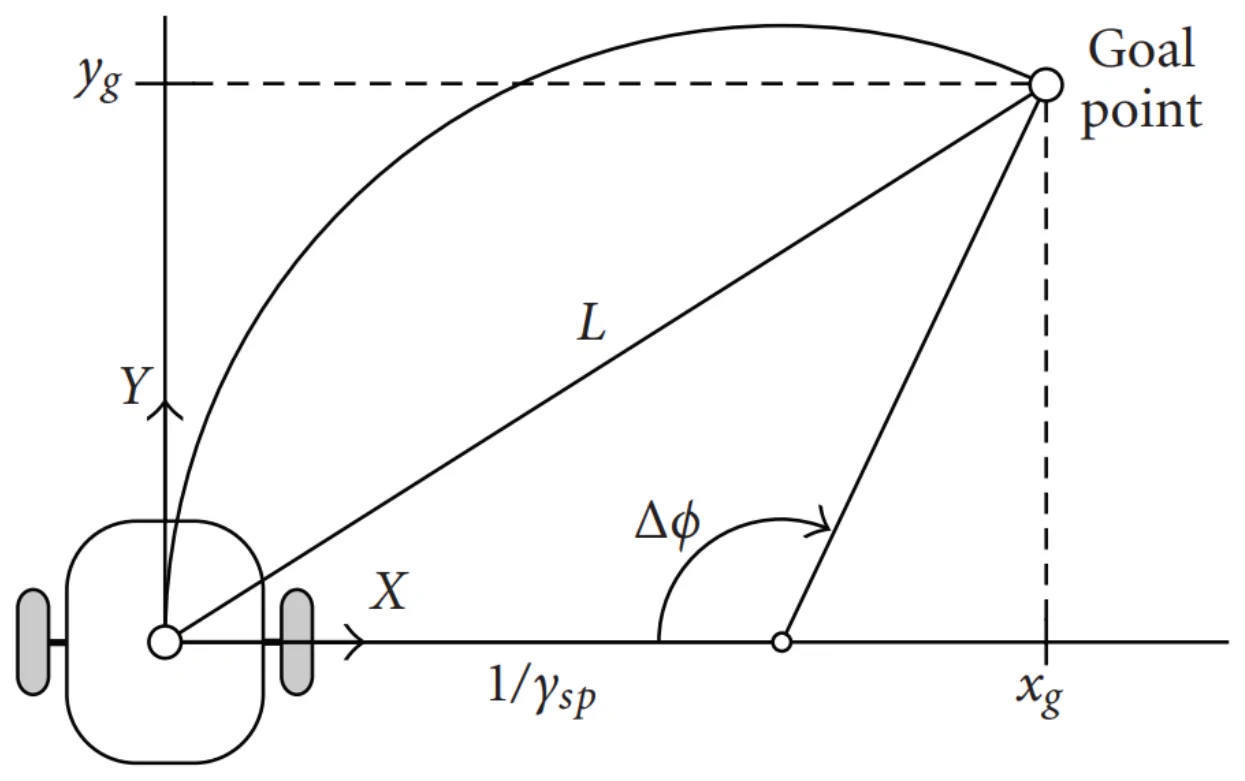
Pure Pursuit Control [79].

The Pure Pursuit Control (PPC) algorithm is a geometric path-tracking method that operates as if the vehicle were chasing a point ahead of it on the predefined path. The controller fits an arc between the vehicle’s current position and a designated lookahead point on the path, located at a predefined distance from the vehicle (the lookahead distance). This arc defines the turning radius, from which the required steering angle to reach the point is computed (Figure 21). The lookahead distance parameter plays a key role in tracking accuracy: the larger the value, the smoother the vehicle’s motion, but the less accurately it follows the path; the smaller the value, the more accurate the tracking, but the more unstable the motion becomes. An advantage of the method is that it is easy to tune and requires simple computations. Pure Pursuit can track not only GPS-based routes but can also process other sensor data; for example, using laser scanner data, it can follow corridors or walls [79].

**Figure 22:**
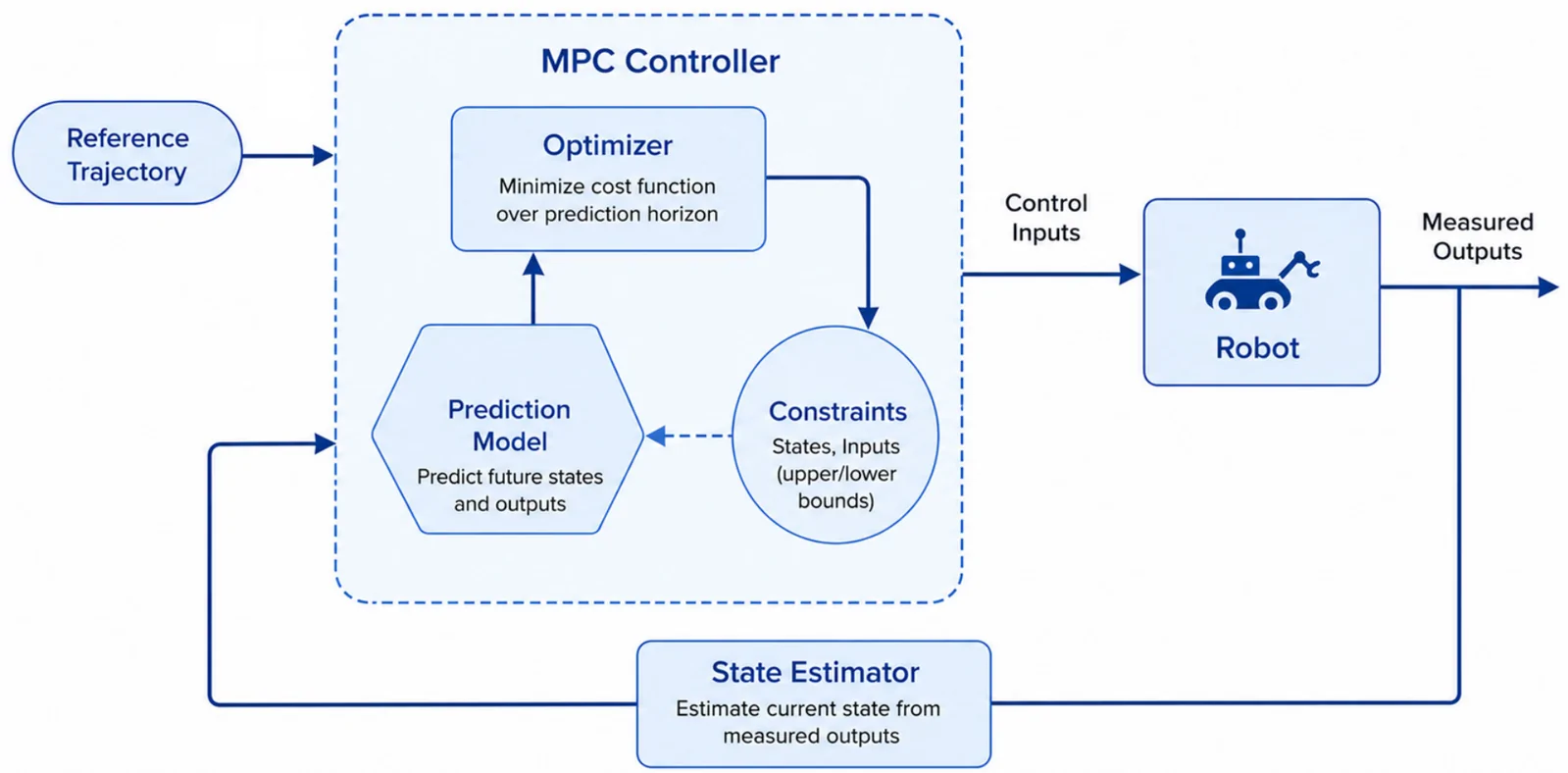
MPC Algorithm. Adapted from [80].

Model Predictive Control (MPC) is a prediction- and optimization-based control strategy. Using a mathematical model of the robot, MPC predicts the system’s behavior over a finite horizon and computes the current control command by minimizing a cost function subject to motion and actuator constraints (Figure 22).

The optimization algorithm receives as input the planned trajectory, the robot’s current position, orientation, and instantaneous velocity, and then computes what path the robot should follow within the given time horizon. It compares this predicted motion to the desired trajectory and, by minimizing the deviation, calculates the appropriate speed and steering angle for path tracking [80,81,82,83].

**Figure 23:**
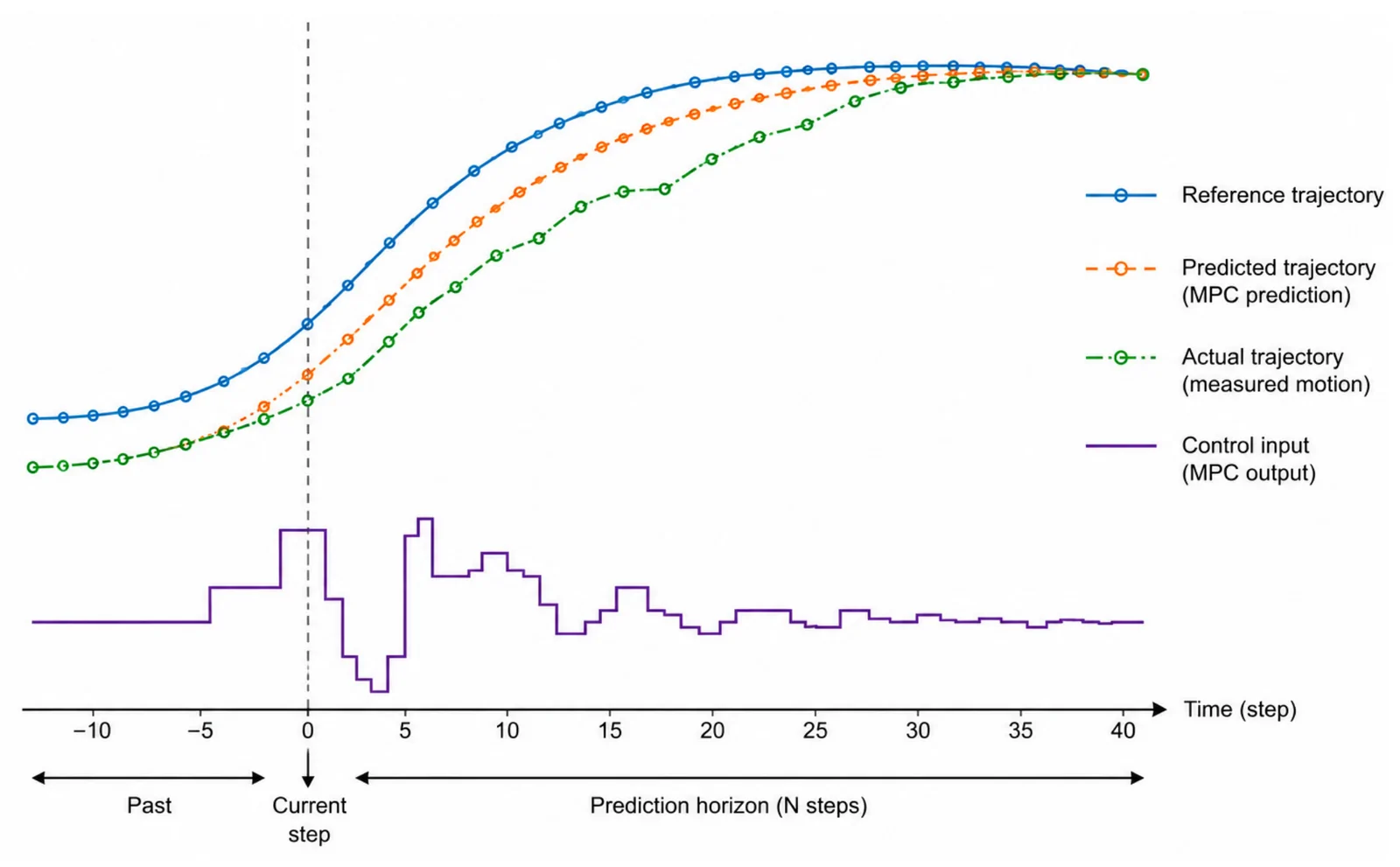
MPC trajectory tracking. Adapted from [80].

Selecting an appropriate prediction horizon is a critical task. If the horizon is too short, the algorithm will not be able to predict far enough ahead, which can make the robot’s motion less accurate (Figure 23). If, on the other hand, the horizon is set too long, the computational demand increases significantly, which can slow execution and result in jerky motion [81,82,83].

MPC is particularly advantageous for agricultural mobile robots because the controller can account for the robot’s operational constraints and uncertainties caused by wheel slip, thereby enabling more accurate path tracking under variable terrain conditions [82,83].

Field experiments have shown that MPC-based controllers consistently outperformed conventional PD-based controllers. An enhanced MPC variant, the Tube-Based Nonlinear Model Predictive Controller (TBNMPC), achieved a performance improvement of 209.72% [82].

MPC can be applied not only to the mobile robot platform but also to manipulator control. During spraying, for example, a common MPC-based controller can coordinate the motion of the mobile platform and the spraying manipulator, ensuring accurate spray application [83].

**Figure 24:**
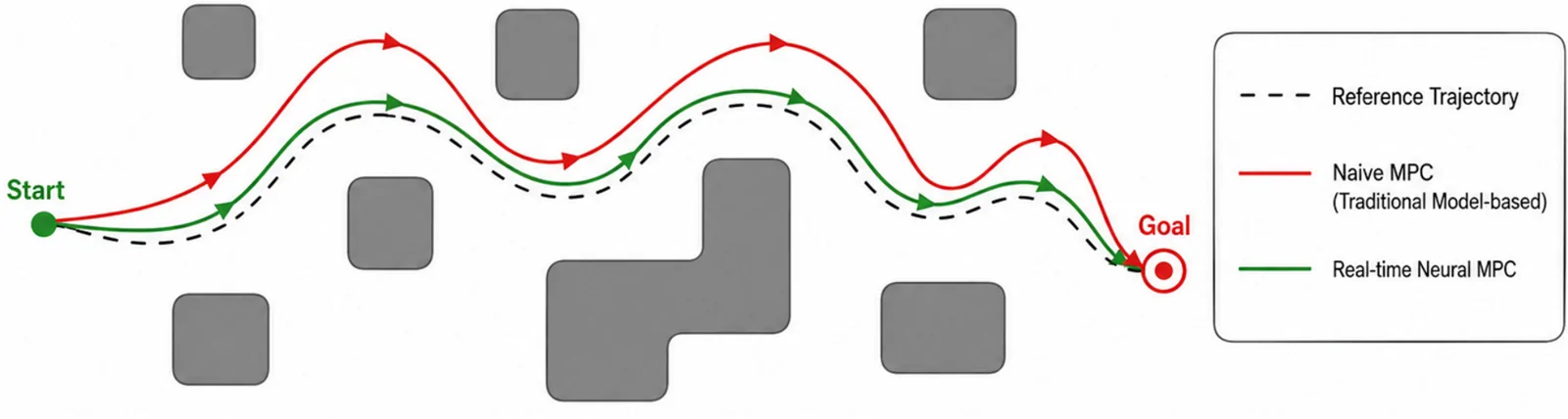
Real-time Neural MPC. Adapted from [84].

MPC-based optimization can be further improved by using machine learning methods—such as neural networks—instead of traditional, static dynamic models. The so-called Real-time Neural MPC approach can describe the system’s complex, nonlinear dynamics more accurately by integrating large-scale neural network models (Figure 24). As a result, higher-precision trajectory tracking can be achieved [84].

**Figure 25:**
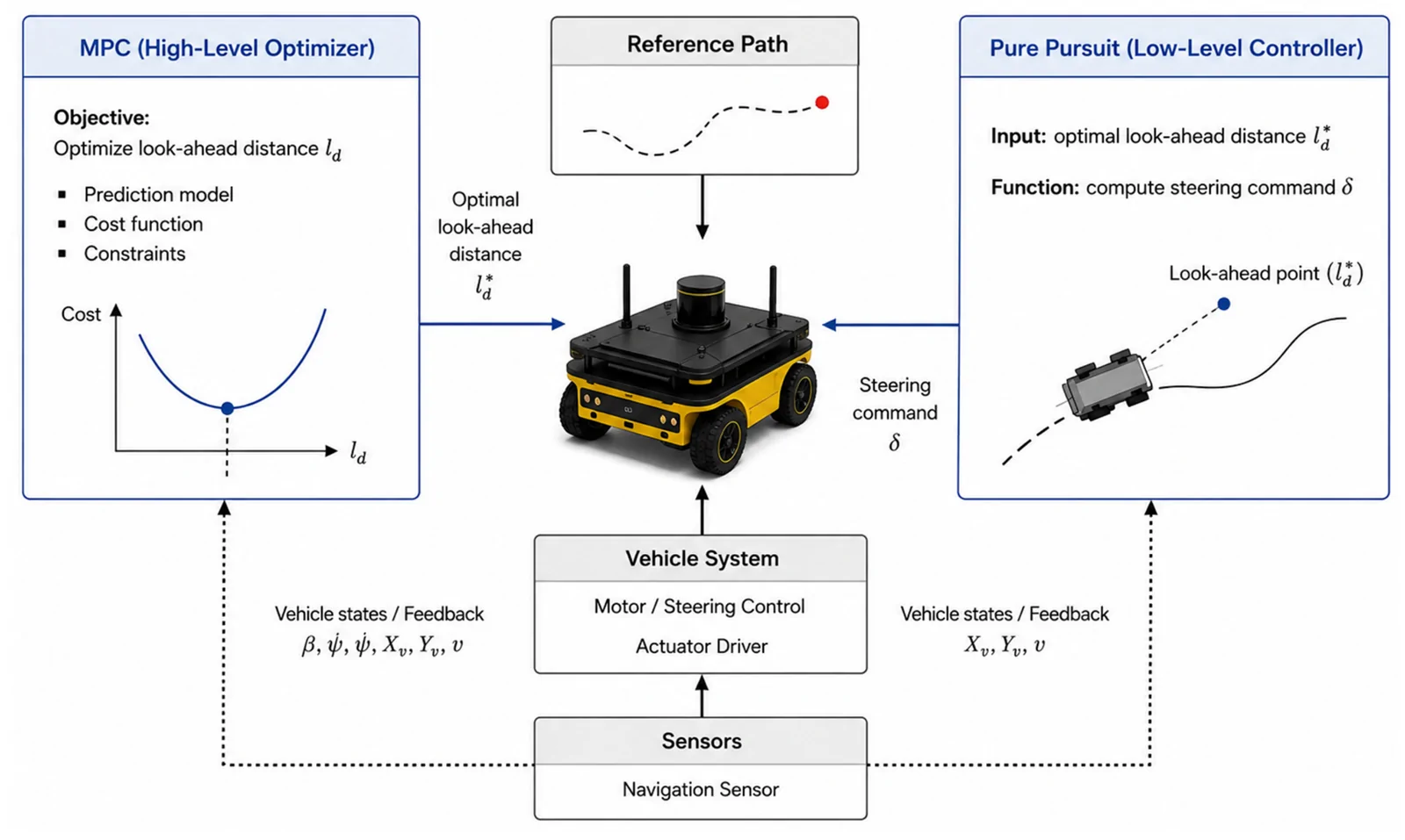
Hybrid MPC-Pure Pursuit control concept. Adapted from [85]. Dotted lines indicate sensor feedback; dashed curves indicate the reference/planned path; solid arrows indicate control signal flow.

The advantages of different motion planners can be integrated into a new hybrid algorithm. For example, the benefits of MPC and Pure Pursuit can be combined by having MPC regulate not the steering angle directly, but the look-ahead distance defined in Pure Pursuit (Figure 25). By default, it sets this value high so that steering remains smooth and natural, and it reduces it only when the optimizer indicates that the vehicle is likely to deviate from the path. Once the risk of deviation has passed, MPC increases the look-ahead distance again up to the allowed maximum, resulting in smoother steering and less jerky motion [85].

In this control architecture, the MPC and PPC algorithms perform different tasks. Pure Pursuit remains responsible for determining the steering command, whereas MPC operates as a higher-level optimization layer. MPC does not directly control the steering angle; instead, it optimizes the look-ahead distance used by the Pure Pursuit algorithm. On straight sections or gentle curves, it selects a larger look-ahead distance, resulting in smoother and more stable steering. For sharp turns or when greater lateral deviation is predicted, it reduces this distance, enabling Pure Pursuit to follow the desired path more quickly and accurately. The advantage of this hybrid approach is that it combines the predictive optimization capability of MPC with the low computational demand of Pure Pursuit, thereby reducing computational cost compared with a fully MPC-based controller.

Table 4 organizes the differences between the motion planning methods described above.

Several strategies are available for obstacle avoidance. The Follow-the-Gap method uses sensor data to identify the largest currently obstacle-free region and steers the robot toward that direction [90].

The Wall Follower algorithm guides the robot along a selected boundary while continuously maintaining a predefined stand-off distance from the wall or crop row [91].

The same families of controllers used for basic trajectory tracking can also be applied to obstacle-avoidance behaviors; for example, a Wall Follower can be implemented with a PID controller [92].

### 4.6. Trajectory Tracking Metrics

Several metrics are used to analyze the efficiency of trajectory planning algorithms. These metrics evaluate both the quality of the generated path and the usability of the algorithm.

When assessing path quality, key considerations include the path length and travel time between the start and goal points. From a safety perspective, collision avoidance and the path–obstacle distance (p–o distance) are important, as they indicate how well the path maintains a safe distance from surrounding obstacles [93].

Various metrics can be applied to examine the shape of a trajectory, such as Euclidean distance, Dynamic Time Warping (DTW), edit distance, and Longest Common Subsequence (LCSS)-based distance measurements [94].

In terms of algorithm applicability, runtime is critical, especially for time-sensitive methods. This can be evaluated using several metrics, such as the solution time measured during individual runs or the ratio of successful solutions within a predefined time limit. Additional important criteria include algorithm stability, which can be assessed via variance across runs, and reproducibility [95].

The ISO 17123-8 standard [96] defines field testing procedures for determining and evaluating the accuracy of GNSS-based field measurement systems, particularly for RTK measurement methods. The standard uses statistical tests to verify that the measured variances meet specified limits. ISO 17123-8 also covers the application of Network RTK (NRTK) solutions [97,98].

The standard distinguishes between two testing procedures: the full and simplified tests. The simplified test consists of a single measurement series aimed at quickly verifying whether the RTK system meets the required accuracy. The full test involves three measurement series, designed to experimentally estimate the variance in determining the position and height of a given point. The error is calculated using the horizontal distance and height difference between two rover points (Equation (5)) [96]:

$$D_{i,j}=\sqrt{{\left(x_{i,j,2}-x_{i,j,1}\right)}^{2}+{\left(y_{i,j,2}-y_{i,j,1}\right)}^{2}}$$

$${\Delta h}_{i,j}=h_{i,j,2}-h_{i,j,1}$$

(5) $$\varepsilon_{D_{i,j}}=D_{i,j}-D^{*}$$

$$\varepsilon_{h_{i,j}}=h_{i,j}-h^{*}$$

i=1,     j=1,…,5

### 4.7. Navigation Errors and Environmental Challenges

Motion planning is one of the most important components of autonomous navigation for agricultural robots. Agricultural environments are characterized by difficult terrain conditions, which pose major challenges for autonomous robots. Dense vegetation can interfere with sensor operation, introducing noise and inaccuracies when estimating the robot’s position [99].

Unlike vehicles operating on roads, agricultural machines typically move on winding, uneven terrain, which makes motion more difficult. Basic motion-planning algorithms generally do not account for the effects of slope and curvature of the terrain, so these approaches cannot be applied effectively in agricultural environments [100].

For agricultural robots to operate truly autonomously, they must be able to recognize dynamically changing environments and guarantee safety for humans, animals, and crops alike. This requires motion planning that can precisely and automatically avoid all obstacles [62].

## 5. Sensor-Fusion Techniques

The complexity of agricultural environments—characterized by loose soil, dense foliage, and unpredictable lighting—means that no single sensor can provide a complete and reliable picture of the robot’s state. Sensor fusion acts as the central processing unit that synthesizes disparate, often noisy data streams into a single, coherent estimation of reality. By leveraging the complementary strengths of different physical modalities (such as the long-range precision of LiDAR and the high-frequency motion tracking of an IMU), these techniques allow robots to maintain spatial awareness even when individual sensors fail or face interference.

### 5.1. Role of Sensor Fusion

For the safe and efficient operation of agricultural robots, a reliable, high-accuracy sensing system is essential—one that can automatically detect and avoid both static and dynamic obstacles such as people, vehicles, vegetation, or other objects. Unlike road-going autonomous vehicles, agricultural robots must not only detect obstacles appearing along a predefined route, but also be able to map traversable—often unknown—areas. In agricultural fields, distinguishing traversable from non-traversable areas is not straightforward. Dense ground cover or tall grass can appear non-traversable even though it may be passable. Conversely, low, flat obstacles such as young seedlings may initially seem traversable, but in reality must be avoided [62].

Sensor fusion is the foundation of reliable and accurate navigation for mobile robots. It combines and jointly processes data from different sensors such as IMU, LiDAR, GPS, UWB, and cameras. Coordinating sensor data within the localization system enables accurate positioning and the estimation of position, orientation, and velocity. Sensor performance can be affected by various environmental factors, leading to measurement errors, which can be compensated for using sensor fusion [101].

The goal of Simultaneous Localization and Mapping (SLAM) is for the robot to determine its own position while simultaneously building a map of its environment. The key idea of sensor-fusion SLAM is to improve localization by integrating data from multiple different sensors [102].

The most commonly used sensors for mobile robots are cameras, LiDAR, IMU, wheel odometry, and GNSS. Cameras and LiDAR sense the robot’s surroundings, and by matching their information, the robot’s motion and position can be estimated. Odometry and the IMU track the robot’s motion. Although these sensors can perform localization on their own, each has limitations; therefore, the most accurate results are achieved through sensor fusion.

SLAM technology is increasingly widely applied in agriculture for autonomous navigation, 3D mapping, field monitoring, and intelligent spraying [103].

A SLAM egy széles körben elterjedt technika, amely a szenzorfúziót használ a lokalizációs teljesítmény javítására.

SLAM is a widely used technique that employs sensor fusion to improve localization performance.

### 5.2. Fusion Methods

The Extended Kalman Filter (EKF) aims to fuse data from the robot’s different sensors (for example, GNSS, LiDAR, and IMU) to provide an accurate estimate of the vehicle’s state and motion. This method is an extension of the traditional Kalman Filter to nonlinear systems, where the nonlinear function is approximated using a Taylor series to obtain a linearized model. EKF consists of two main steps: prediction and update. First, it predicts the next state based on the robot’s previous motion. Then, it corrects this estimate using new sensor measurements. EKF increases the update frequency of position estimates and smooths discrepancies between data streams from different sensors, thereby improving localization accuracy [104].

The Unscented Kalman Filter (UKF) is, similar to EKF, an algorithm developed for nonlinear systems. Unlike EKF, it does not use linear approximations; instead, it employs sigma-point-based statistical sampling via the Unscented Transform (UT) to estimate the system state. Around the estimate, it approximates the true distribution using a Gaussian distribution, thereby increasing estimation accuracy [105].

**Figure 26:**
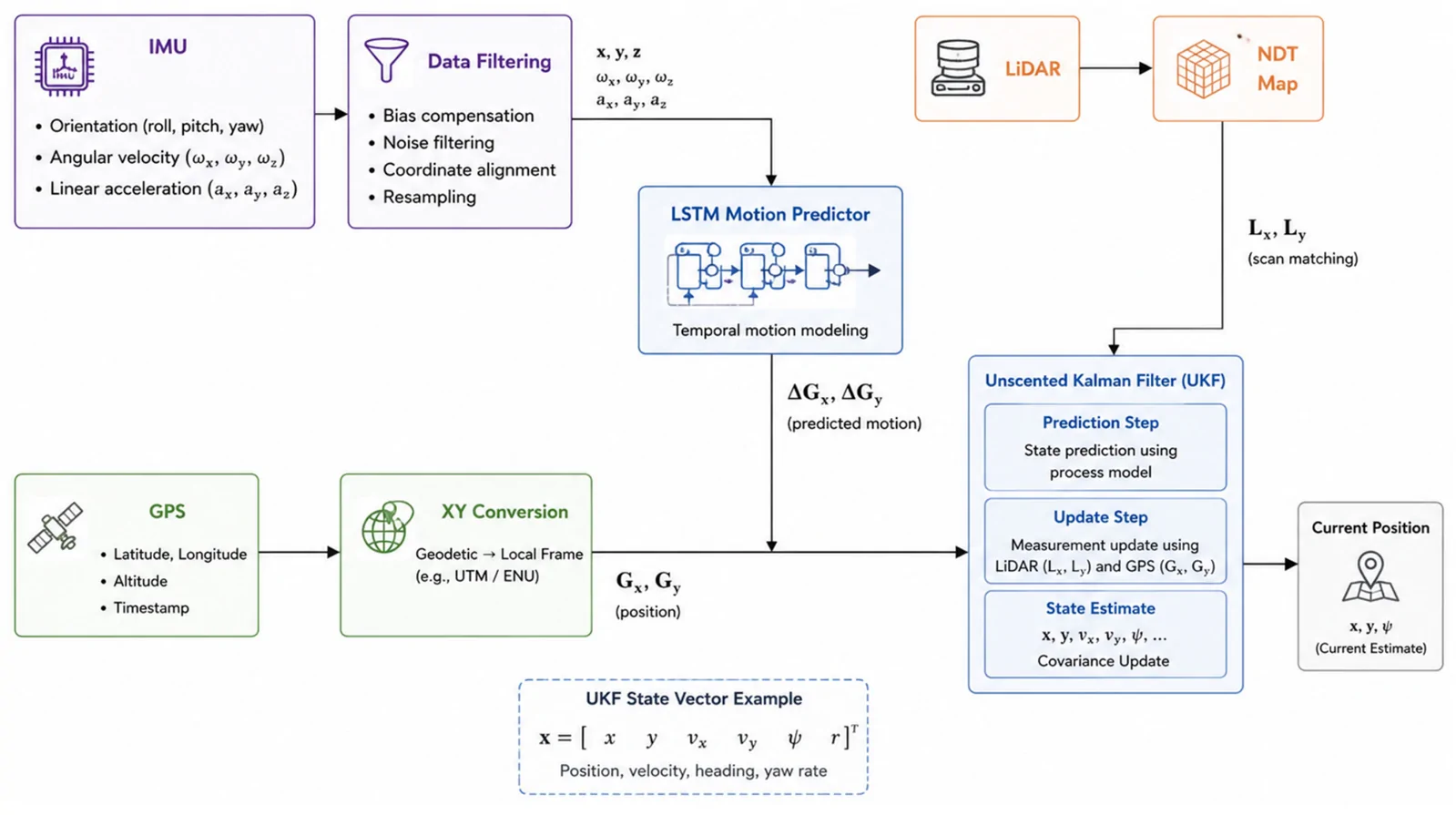
Unscented Kalman Filter. Adapted from [105].

Figure 26 illustrates the UKF-based sensor-fusion process, which determines the robot’s current position by combining orientation data from the IMU sensor, position data from the GPS, and environmental information from the LiDAR.

The Adaptive Neuro-Fuzzy Inference System (ANFIS) is a sensor-fusion method that combines the decision-making flexibility of fuzzy logic with the learning capability of neural networks. This enables the system to learn from data and continuously improve the accuracy of position estimation. Training the model requires collecting a large amount of data so that it can adapt to different localization conditions. ANFIS provides more accurate localization than EKF [106].

In study [106], ANFIS- and EKF-based sensor-fusion methods were compared using an outdoor mobile robot, as summarized in the new Table 5. The mean localization error was 2.58% for ANFIS and 5.67% for EKF, corresponding to an approximately 54.5% reduction in mean error with ANFIS. In addition, the standard deviation of the ANFIS errors (3.09%) was lower than that of the EKF errors (13.31%). This indicates more stable localization performance under the conditions of that experiment.

**Figure 27:**
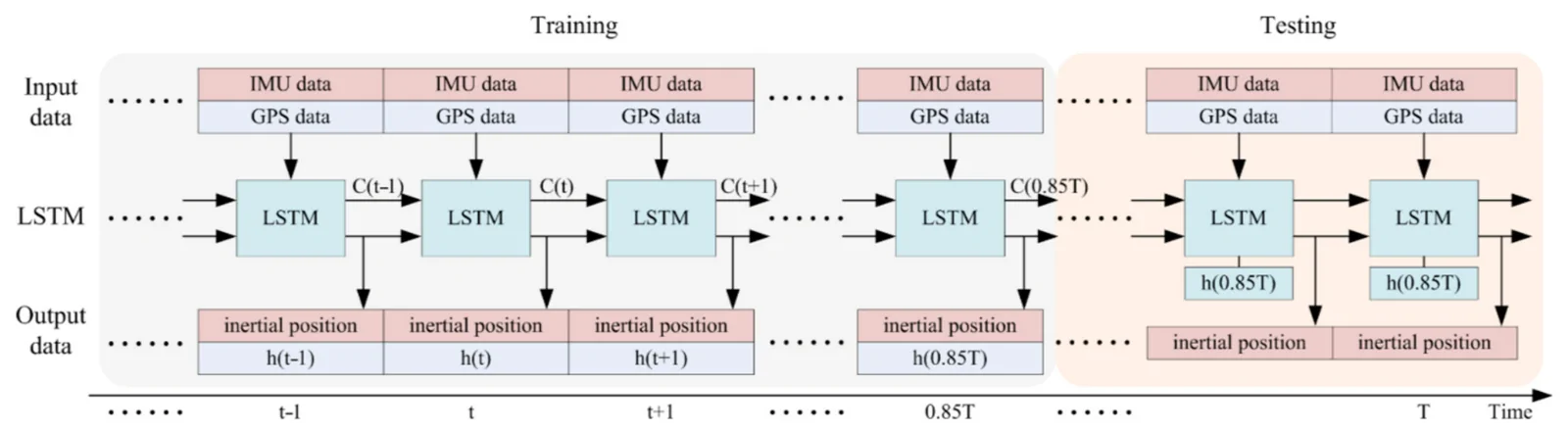
LSTM [107].

Kalman Filter-based solutions improve the accuracy of position estimation, but they cannot completely eliminate drift originating from IMU and GPS sensors. Applying deep learning methods makes nonlinear system modeling more effective and can significantly reduce drift. An LSTM (Long Short-Term Memory) neural-network-based sensor-fusion approach continuously integrates IMU and GPS data for position estimation. The LSTM learns temporal patterns in the data, making it well suited for systems where past states influence future behavior (Figure 27). The model consists of four main components (forget gate, input gate, candidate layer, and output gate), which together ensure that the model selectively retains or forgets prior information [107].

Table 6 summarizes the main characteristics of the investigated sensor-fusion methods.

**Figure 28:**
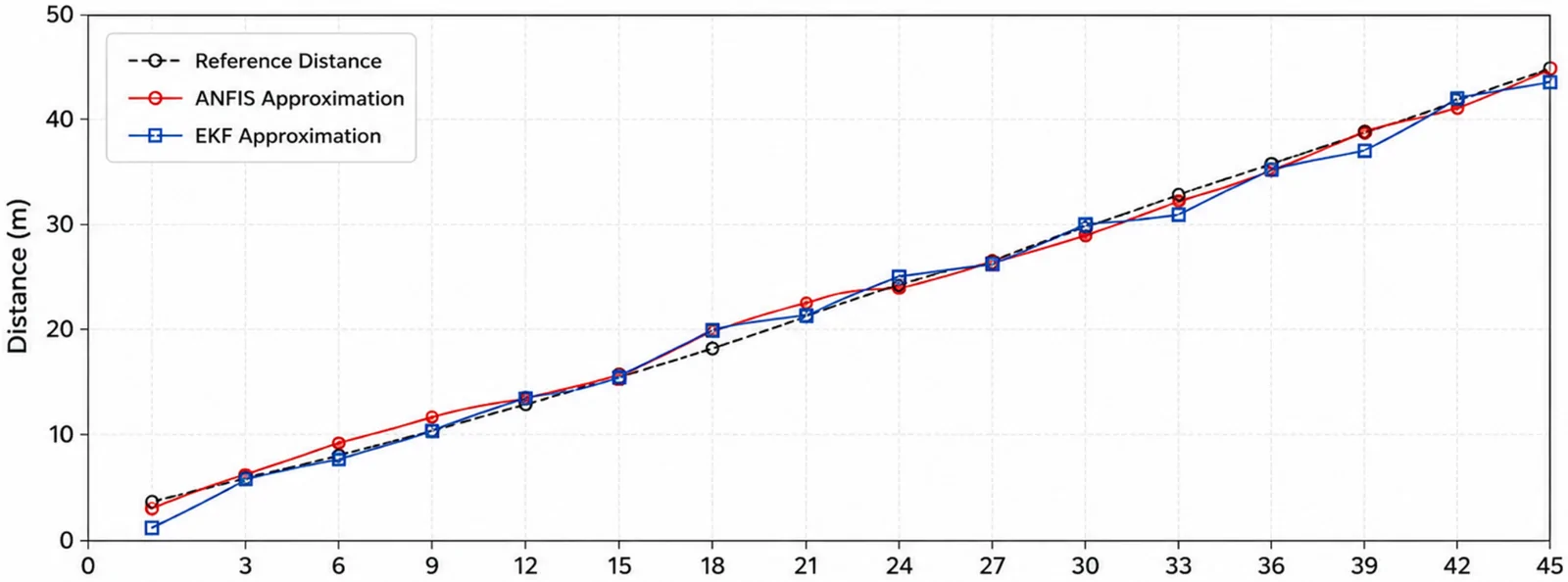
Comparison of ANFIS and EKF. Adapted from [105].

As summarized in Table 6, filter-based methods (EKF/UKF) remain attractive because of their efficiency and relative ease of deployment, whereas learning-based fusion and multi-sensor SLAM can improve robustness in nonlinear or drift-prone conditions at the cost of greater data, tuning, and computational requirements (Figure 28).

Particle Swarm Optimization (PSO) is a population-based metaheuristic optimization technique inspired by the collective movement of bird flocks and fish schools. Rather than acting as a standalone state estimator, PSO is typically applied to tune the parameters of the fusion algorithms discussed above—for example, optimizing the process- and measurement-noise covariance matrices of the EKF/UKF, or the membership-function parameters of ANFIS—by iteratively updating a swarm of candidate solutions based on their own and the swarm’s best-known positions. This approach can reduce the manual tuning effort associated with filter-based methods and has been applied in agricultural robotics to improve localization accuracy under variable field conditions, though it adds computational overhead during the (typically offline) tuning phase [108].

### 5.3. Role of Real-Time Synchronization and the ROS Ecosystem

Accurate time synchronization of sensor data is crucial for reliable sensor fusion. Different sensors on robots (for example, GPS and IMU) operate as independent units. Clock offsets between sensors and data transmission delays can cause temporal misalignment errors. If data arrive at different times, fusion algorithms may produce incorrect estimates. Both hardware- and software-based solutions exist for time synchronization [109].

Several middleware frameworks are available to support communication among hardware units, including YARP, OROCOS, MOOS, and ROS. Some of these middleware systems focus primarily on specialized application domains: YARP is used mainly in humanoid robotics, whereas MOOS targets marine robotics. OROCOS and ROS were developed to support general-purpose robotics; however, ROS has become the most widely used middleware framework. Consequently, it offers substantially broader hardware and driver support, an extensive software-package ecosystem, and an active developer community [110].

ROS 2 was developed as an evolution of the original ROS and now has even broader support. The first alpha version of ROS 2 was released in 2018, and several Long-Term Support (LTS) and interim releases have appeared since then. Multiple distributions are currently under active support. Lyrical Luth, released in May 2026, is the latest LTS release; the non-LTS Kilted Kaiju and the LTS Jazzy Jalisco and Humble Hawksbill releases also remain actively supported.

The Lyrical Luth release primarily introduced performance improvements, better CPU utilization, and enhanced memory management. In addition, the AsyncNode class was officially introduced and integrated, supporting parallel data processing tasks such as sensor handling [111].

Before AsyncNode was introduced, the ROS 2 community had already made several attempts to integrate Python (version 3.X) asyncio with ROS 2. Although these solutions enabled asynchronous task management, they were generally not official components of ROS 2 [112].

The Robot Operating System (ROS) is an open-source middleware that supports the development and communication of robotic systems through its modular architecture. The system is built from independently operating nodes that exchange data via topics using the publisher/subscriber paradigm. One node publishes data, and another subscribes to it and processes the messages [113].

**Figure 29:**
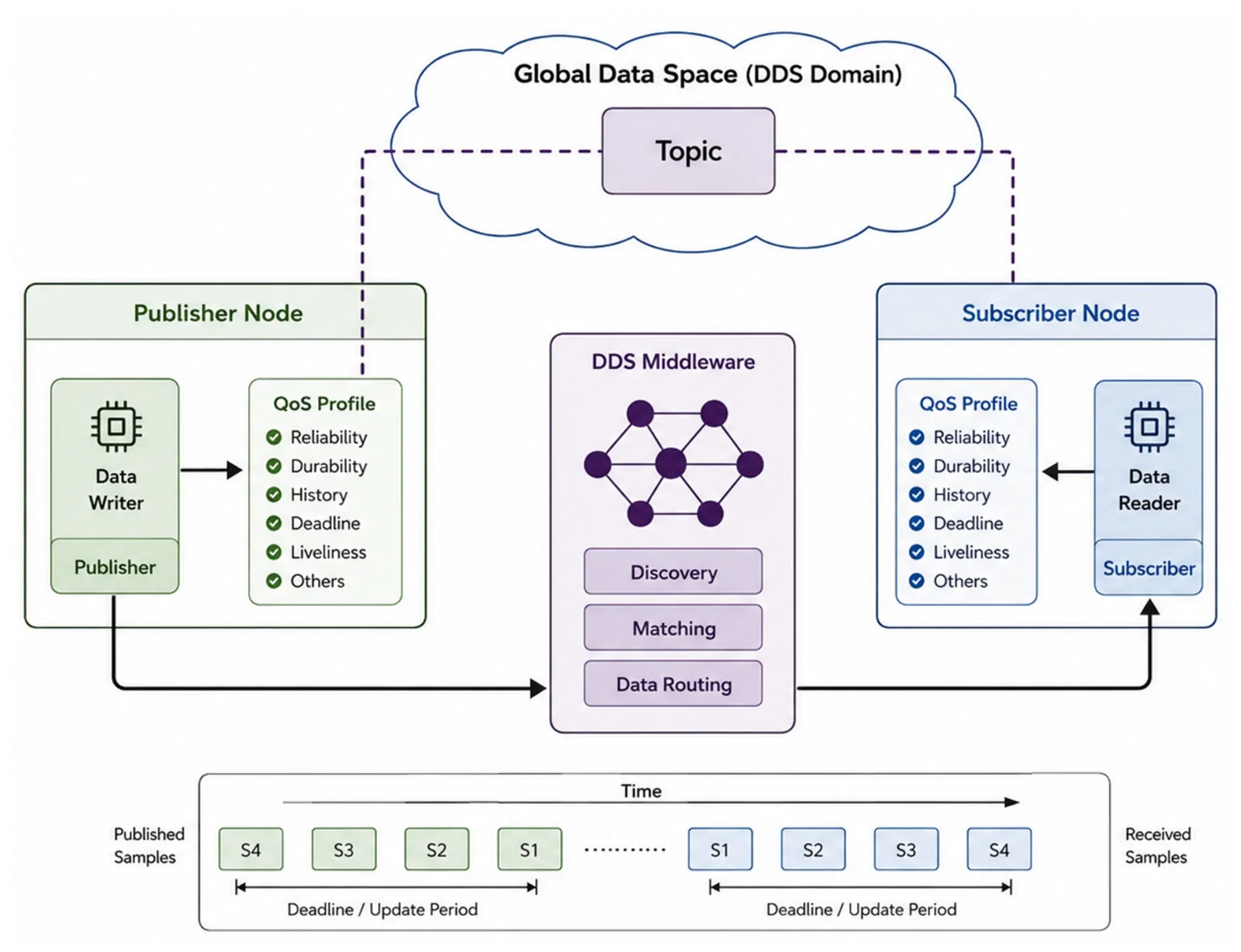
DDS. Adapted from [113].

Communication in ROS 2 is provided by the Data Distribution Service (DDS), which was designed to meet the needs of real-time systems. DDS uses a publisher/subscriber communication model, made more reliable through Quality of Service (QoS) settings. (Figure 29). QoS parameters define the characteristics of data transmission and the scheduling of data updates so that information arrives on time [114].

The ROS 2 tf2 transformation library manages conversions between the coordinate frames used by the robot’s various sensors. tf2 links the robot’s modules and the reference frames of sensors and other components into a tree structure. This enables fast and accurate transformations between any two coordinate frames. tf2 supports coordinate transformations—both rotations and translations—in both static and dynamic frames [115].

**Figure 30:**
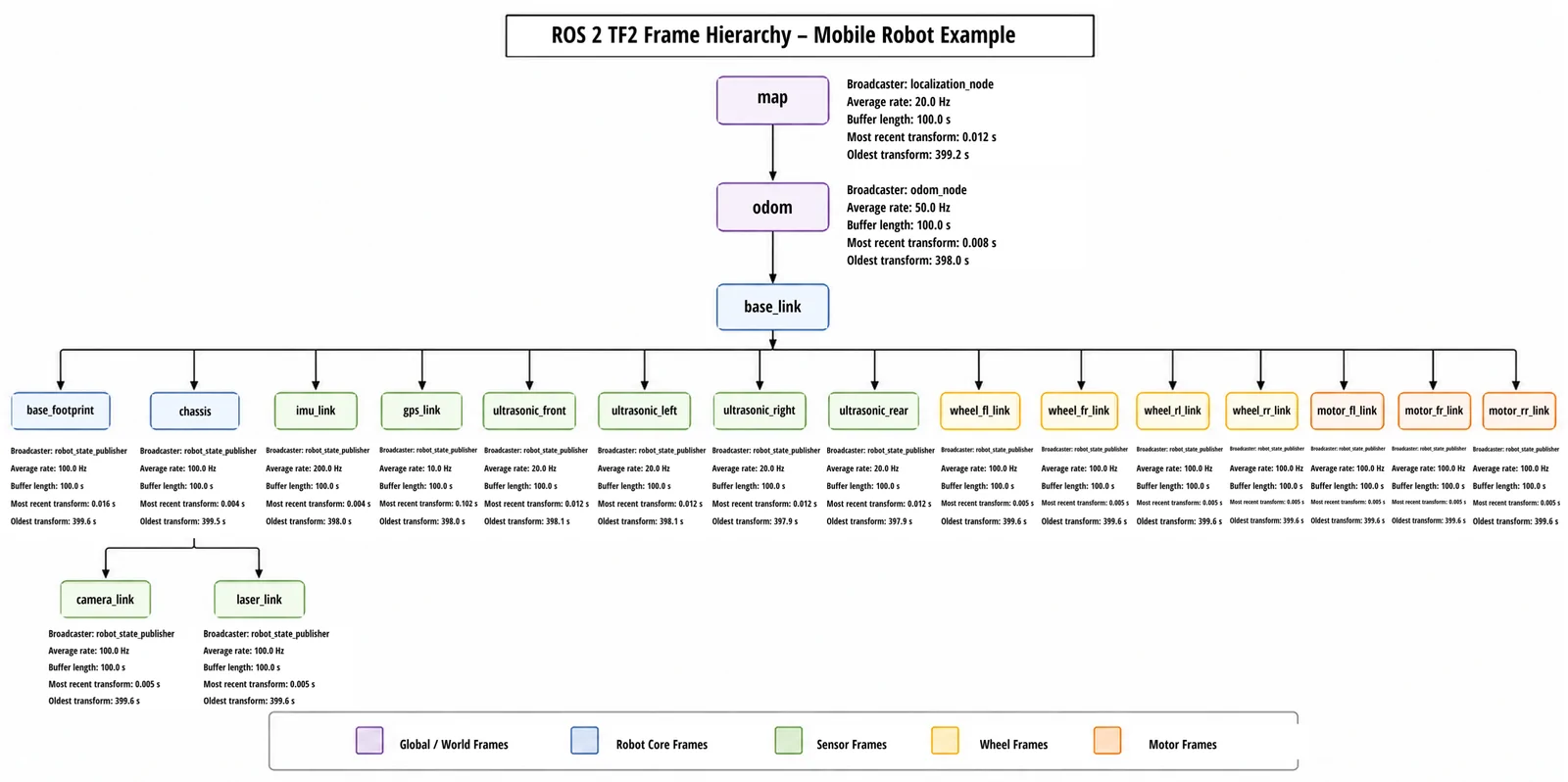
TF2 coordinate-frame hierarchy. Adapted from [115].

Figure 30 illustrates the TF2 coordinate frame hierarchy used in the ROS 2 ecosystem. It shows a possible TF2 transformation chain, where the odom frame serves as the global reference, and base_link acts as the central coordinate frame, to which the coordinate frames of the various sensors are attached.

## 6. Communication and Data Management

The transition from isolated autonomous machines to integrated smart farming ecosystems relies heavily on the seamless flow of information. Modern agricultural robots are no longer standalone units; they function as mobile edge-computing nodes that must communicate with cloud interfaces, remote operators, and other autonomous agents.

### 6.1. Internet of Things (IoT)

The Internet of Things (IoT) is a system of networked physical devices in which uniquely identifiable electronic devices can communicate and cooperate over a network. These devices may include embedded systems equipped with sensors and actuators that are capable of collecting and transmitting data. The IoT is founded on the integration of technologies that provide distributed intelligence [116].

In recent years, the IoT has become one of the most rapidly developing areas of information technology and telecommunications. Its development is driven by the continuously growing demand for automated data collection and processing. The IoT supports real-time monitoring and intelligent decision-making. The technology is now used in nearly every field, including precision crop production.

The rapid development of the IoT has been supported by advances in smart devices, wireless communication technologies (Wi-Fi, Bluetooth, ZigBee, NFC, and 5G), cloud computing, big-data analytics, and artificial intelligence. All of these technologies are essential to IoT operation.

The original IoT architecture consists of three layers. The Perception layer observes the physical environment, where sensors collect data. The Network layer is responsible for applying different communication technologies and transmitting the data appropriately. The Application layer transforms the collected information into specific services.

However, as the application areas and functionality of the IoT have continued to expand, the initial architecture has increasingly been replaced by a seven-layer architecture.

In the new model, the Physical Devices and Controllers layer comprises the physical devices and sensors themselves; the Connectivity layer provides network connections among devices; the Edge Computing layer performs time-critical data processing locally; the Data Accumulation layer collects and stores incoming data; the Data Abstraction layer standardizes and prepares the data for further processing; the Application layer implements the various IoT applications and services; and the Collaboration and Processes layer integrates decision-support systems [117].

The IoT is a fundamental technology in precision crop production, providing real-time information about field and crop conditions. IoT sensors can continuously monitor soil moisture, temperature, humidity, pH, light conditions, and crop status. The collected data can then be analyzed automatically, enabling irrigation, nutrient management, and crop protection to be optimized while reducing the use of water, fertilizers, and other resources and increasing production efficiency.

IoT technology is closely linked to precision agricultural robots and automated systems. Through IoT networks, robots and sensors collect and transmit environmental data in real time, enabling robots to autonomously perform site-specific interventions [118].

The open-source FIWARE (Future Internet WARE) platform supports the development of IoT-based applications by providing standardized services for cloud-based data management, storage, and sharing. ROS-based robotic systems can connect to FIWARE through FIROS or a FIWARE-ROS bridge, enabling data exchange between robots and IoT devices, access to data stored in the cloud, cloud-based data processing, and robot supervision [119].

### 6.2. Role of 5G and MQTT

5G technology plays a key role in mobile robotics because it can provide real-time, highly reliable data transmission. Real-time processing is especially important for navigation and localization tasks, where fast processing of LiDAR, camera, and other sensor data—as well as efficient communication between robots—is essential for safe operation. 5G enables mobile robots to connect directly to cloud-based systems, providing access to the high-compute resources required by artificial intelligence and machine learning algorithms. This makes it possible to solve tasks that exceed the computing capacity of the onboard control PC [120].

However, safety-critical functions such as obstacle avoidance and basic navigation cannot rely exclusively on 5G connectivity or cloud-based processing. Therefore, the robot must be capable of autonomous navigation even without a network connection, although potentially with reduced functionality.

MQTT is a publisher/subscriber-based communication protocol developed for IoT devices. It is ideal for data transfer over low-bandwidth, unstable, or remote networks, as it provides reliable and robust operation even under unfavorable network conditions. A central element of the MQTT architecture is the broker, which acts as a message distributor between clients (publishers and subscribers). MQTT has a simpler structure than ROS, so it can be deployed more quickly; however, unlike ROS, it is intended only for message passing. The protocol offers simple message structures, good error handling, and reliability, making it well suited for basic communication tasks, monitoring, and data collection. In addition, it can serve as a communication bridge between different devices or systems [121].

### 6.3. Big Data Collection and Cloud-Based Processing

Establishing sustainable agriculture is a data-intensive process in which data collected by various sensors must be managed jointly. Introducing Big Data technologies into agriculture supports the development of smart farming. It helps optimize irrigation, crop protection, and harvesting, enabling more efficient resource use with a lower environmental burden. The application of AI and machine learning further supports data processing and decision-making, as these methods can identify patterns and relationships within the data [122].

Time-series data collected by agricultural machinery and sensors provide a spatiotemporal picture of farmland. In precision agriculture, these data can support yield forecasting, weather-aware decision-making, and risk monitoring. Cloud-based processing enables large, heterogeneous datasets to be integrated and analyzed using machine-learning methods such as KNN, SVR, decision trees, and Random Forests. Among these, Random Forest regression is often reported as a strong baseline for crop-yield prediction because it can handle nonlinear relationships and heterogeneous inputs with limited feature engineering [123,124].

Random Forest is a classification and regression algorithm that builds multiple decision trees and produces the final prediction based on their averaged output or majority vote. Its purpose is to increase accuracy and avoid overfitting (learning the noise and errors in the training data). Each decision tree is trained on different samples and randomly selected features, so they learn independently from one another. This built-in randomness reduces correlation between trees, making the model more stable and producing more reliable predictions [124].

### 6.4. GUI and Real-Time Visualization Options

RViz is a 3D data visualization tool widely used in robotics for analysis, testing, debugging, research, and demonstration tasks. RViz can display information from various sensors and other data sources in real time within a unified environment. It supports visualization of data from cameras, LiDAR sensors, sonar, GPS, and other sensors. It also enables the combined display of information from the robot’s navigation and control modules, helping to track the robot’s operation and behavior in real time.

RViz provides a flexible, customizable interface for different visualization needs. It includes many display types such as 3D models, point clouds, laser scan data, and heatmaps, and it also allows visualization of the robot’s kinematic and trajectory data as well as the creation of interactive markers and menus. RViz is tightly integrated with the ROS ecosystem [125,126].

**Figure 31:**
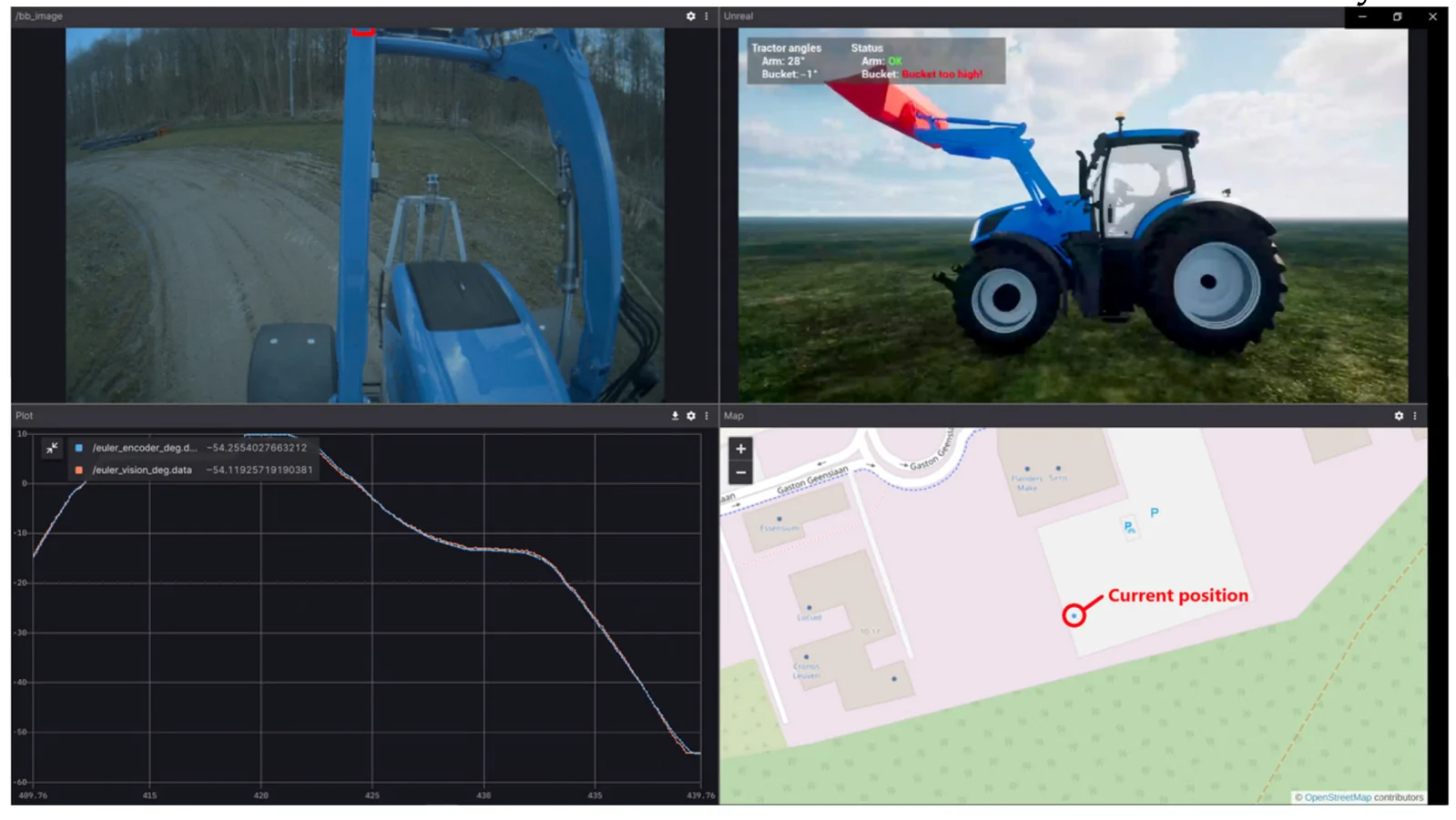
Foxglove panel [127].

Foxglove Studio is a data visualization platform that enables real-time display of various sensor data and system information on a single, clear dashboard. It allows multiple views to be combined within one interface. For example, you can simultaneously see the live feed from an onboard camera, a virtual 3D model of the vehicle, and charts visualizing different computed values. In addition, an OpenStreetMap-based map can be displayed that tracks the robot’s movement using its GPS data (Figure 31). Foxglove supports ROS connections, so it can receive and visualize real-time data directly from ROS nodes [127].

## 7. Digital Twin Concept for Robot Testing

A new area in robot testing is the application of the digital twin concept. There are three levels of modeling: the “digital model,” “digital shadow,” and “digital twin pair.” In the case of a digital model, there is only a static correspondence between the real object and its 2D or 3D model—meaning that changes in the real object are not reflected by changes in the model. In the case of a digital shadow, an (updating) copy of the physical object is essentially created, but the correspondence is one-way: any change in the digital copy does not affect the real object. In contrast, in the case of a digital twin pair, a true two-way connection is established between the physical and virtual counterparts [128,129].

### 7.1. Legal Requirements

Regulation (EU) 2023/1230 lays down safety requirements for the operation of machinery and extends its scope to machines using new digital technologies. It pays particular attention to machines equipped with IoT and artificial intelligence, as well as robots, taking into account the new safety risks associated with these technologies. The primary aim of the regulation is to ensure user safety; therefore, it sets mandatory requirements, such as the use of protective devices and emergency stop equipment [130,131].

### 7.2. Simulation Platforms

Simulation plays a central role in the development of agricultural robots because development and testing in real environments require substantial time, cost, and equipment. Virtual environments allow complex agricultural workspaces and robot behavior to be modeled realistically, enabling different control algorithms and navigation strategies to be tested and optimized safely. Simulation facilitates software debugging, coordination of hardware and software components, and comparison of the performance of different software solutions [132].

In real environments, certain hardware configurations may create hazardous situations for operators and crops. Therefore, the robot’s operation should initially be evaluated in simulation, where control algorithms can be developed and tested without risking damage to the physical robot or agricultural equipment. For this purpose, the simulation must realistically model characteristics of the agricultural environment, including terrain, soil interactions, weather conditions, and crop stands, because these factors substantially affect the operation of autonomous robots [133].

Several simulation platforms are available, but Gazebo is currently one of the most widely used open-source robotics simulators. It is developed by the Open Robotics Foundation, the same organization that coordinates ROS development. Gazebo can therefore be regarded as the primary simulation environment with native ROS support. Its close integration with ROS allows developers to use the same ROS packages, nodes, topics, services, and algorithms in simulation as on physical robots, substantially facilitating transfer of developments to real hardware.

One of Gazebo’s most important features is its high degree of modularity and customizability, which is also characteristic of the ROS environment. The mechanical structure of robots can be defined using Unified Robot Description Format (URDF) files, which contain the robot’s geometric model, kinematics, mass and inertia parameters, and the properties of individual motors and sensors. This enables realistic modeling of different mobile robots.

As a general-purpose simulation platform, Gazebo supports numerous robotics applications, including SLAM [134,135,136].

Gazebo requires complex configuration and substantial computational resources. As an alternative, the ROS-compatible MAES (Multi-Agent Exploration Simulator) was developed. MAES is not a general-purpose simulator; rather, it is optimized for the narrower application domain of exploration and mapping [136].

Gazebo is also suitable for implementing a digital twin because it can create a real-time virtual replica of the physical robot and provide continuous data exchange between the physical and virtual systems through the ROS communication layer [137].

## 8. Challenges, Deployment-Oriented Design Guidance, and Future Directions

From a deployment perspective, no single navigation stack dominates all agricultural settings. In open, structured fields with stable GNSS reception, RTK GNSS fused with IMU and wheel odometry remains a practical baseline for global localization, while LiDAR- or camera-assisted local perception is needed for row following and obstacle avoidance [52,62,104]. In orchards, vineyards, and other GNSS-challenged environments, multi-sensor SLAM and tighter LiDAR/vision/IMU fusion become more attractive, despite higher compute and integration effort [102,103]. For planning and control, map-based global planners such as Dijkstra or A* are suitable when a stable field map is available, whereas DWA, TEB, and related local planners are necessary when vegetation, workers, or machines introduce dynamic obstacles [60,63,72]. Lightweight controllers such as PID and Pure Pursuit remain attractive for low-cost platforms, while MPC is preferable when curvature constraints, actuator limits, and multi-objective optimization must be handled explicitly [77,79,81].

PID controllers are simple and effective but require accurate parameter tuning. Pure Pursuit has low computational demand, but its accuracy is lower than that of MPC. MPC can account for vehicle dynamics and system constraints, but it requires greater computational capacity [77,79,81].

Across these configurations, field robustness depends not only on the core algorithm but also on engineering discipline at the system level. Accurate time synchronization, ROS 2/DDS communication design, TF management, safety layers, and reproducible validation protocols are prerequisites for reliable autonomy rather than optional implementation details [98,113,114]. Future research should therefore prioritize benchmark datasets from real agricultural environments, cross-season evaluation, explicit reporting of compute and energy budgets, GNSS-outage robustness, crop-safety metrics, and digital-twin workflows that support repeatable test campaigns before field deployment [95,128,129]. In parallel, modular architectures, edge–cloud integration, and sustainability-aware design will be important for scalable smart-agriculture robots that can move from laboratory prototypes to dependable on-farm systems [120,138,139].

## Figures and Tables

**Table 1:** Total Number of Search Results Returned by Each Database.

Database	Robot	Agricultural Robot	Field Robot	Precision Agriculture
Scopus	519,375	55,049	68,493	41,113
Web of Science	256,485	3268	264	20,791
IEEE Xplore	235,530	3277	42,183	16,721
ScienceDirect	341,158	32,324	202,739	199,704
SpringerLink	182,278	7927	99,049	85,817
MDPI	23,223	987	3855	6762

**Table 2:** Relative Annual Distribution (%) of Search Results.

Year	Robot (%)	Agricultural Robot (%)	Field Robot (%)	Precision Agriculture (%)
2026	8.42	7.92	13.03	19.20
2025	13.38	10.38	15.65	21.94
2024	11.93	7.68	13.08	13.85
2023	10.02	5.74	10.33	8.84
2022	9.60	5.63	9.36	7.50
2021	9.99	21.19	9.08	6.43
2020	9.16	23.53	7.81	5.41
2019	7.28	5.79	5.69	4.57
2018	6.29	2.85	4.75	3.80
2017	5.76	2.39	4.12	3.21
2016	4.15	1.74	3.65	2.75
2015	4.01	5.17	3.45	2.49

**Table 3:** Comparison of trajectory planning algorithms (global vs. local) [69,70,71,72,73,74,75].

Algorithm	Planning Level	Dynamic Obstacle Avoidance	Path Smoothness	Suitability for Precision Mobile Robots	Typical Representation	Environment Representation (Typical Data Sources)	Computational Cost (Typical)
Dijkstra	Global	Only through replanning	Grid-based	Partially suitable	Weighted graph	Map-based (e.g., GNSS/IMU/odometry-derived map)	O((V + E) log V) (with priority queue); often reported as O(n log n)
A*	Global	Only through replanning	Piecewise linear (with waypoints)	Partially suitable	Weighted graph + heuristic (typically grid/occupancy grid)	Map-based (e.g., GNSS/IMU/odometry-derived map)	Typically O((V + E) log V); worst-case can degrade toward exponential depending on heuristic/branching
DWA	Local	Suitable	Smooth	Suitable	Velocity space sampling (v, ω)	Online perception (e.g., LiDAR/camera/ultrasonic + IMU)	O(Nv·Nω·Nh) (samples × horizon discretization); often summarized as O(n^k)
TEB	Local	Suitable	Smooth	Suitable	Time-parameterized trajectory optimization	Online perception (e.g., LiDAR/camera/ultrasonic + IMU)	Often summarized as O(k·n) (k iterations, n trajectory states/po

**Table 4:** Comparison of motion planning algorithms [86,87,88,89].

	PID	MPC	PPC
Control method	Feedback-based	Predictive	Look-ahead
Objective function	Error-based	Optimization of future horizons	Path geometry curvature calculation
Terrain	Environment-independent	Environment-independent	Requires known reference path
Accuracy	Good (tuning-dependent)	Very good (model-dependent)	Medium (curvature-dependent)
Robot model	No model required	Detailed model	Simplified model
Obstacle avoidance	Requires replanning	Supported	Requires replanning
Suitability for precision mobile robots	Partially suitable	Suitable	Partially suitable

**Table 5:** Comparison of ANFIS and EKF performance [106].

Reference (m)	ANFIS (m)	EKF (m)	ANFIS (%Error)	EKF (%Error)
3	3.301841434	1.398909494	10.061381	53.369680
6	6.338048882	6.152553211	5.634148	2.542554
9	9.683320755	8.735862053	7.592453	2.934866
12	12.490936770	12.607932170	4.091140	5.066101
15	15.181936960	15.095180360	1.212913	0.634536
18	18.546405560	19.140863770	3.035586	6.338132
21	21.763895490	21.592793410	3.637598	2.822826
24	24.211553270	24.909966760	0.881472	3.791528
27	26.993683610	26.941816070	0.023394	0.215496
30	30.176623150	30.489224560	0.588744	1.630749
33	32.929363880	32.526966840	0.214049	1.433434
36	36.119310530	36.067254310	0.331418	0.186818
39	39.437282690	38.271436490	1.121238	1.868112
42	41.889555220	42.459950990	0.262964	1.095121
45	44.976040130	44.490193380	0.053244	1.132904

**Table 6:** Comparison of sensor-fusion and localization approaches for autonomous field robots in precision agriculture.

Method	Typical Fused Inputs	Primary Output	Strengths (Field-Relevant)	Limitations/Risks	Compute/Implementation Notes
Multi-sensor SLAM (fusion-based SLAM)	Camera and/or LiDAR + IMU + wheel odometry (+GNSS when available)	Pose + map (often 3D)	Improves localization by integrating multiple sensors; increasingly used in agriculture for autonomous navigation, 3D mapping, monitoring, and intelligent spraying [102,103].	Performance can degrade in highly dynamic vegetation and repetitive structures; tuning and initialization can be non-trivial [102].	Compute depends on sensing modality and backend; typically heavier than filtering approaches [102].
EKF	GNSS + IMU (+LiDAR)	State estimate (pose, orientation, velocity)	Increases the pose update frequency and smooths discrepancies across sensor streams, improving localization accuracy [103].	Linearization via Taylor approximation can reduce accuracy in strongly nonlinear regimes [104].	Relatively lightweight, but sensitive to process/measurement noise tuning [104].
UKF	GNSS + IMU (+LiDAR)	State estimate (pose, orientation, velocity)	Handles nonlinearities without explicit linearization using sigma-point sampling (Unscented Transform), improving estimation accuracy [105].	Higher computational demand than EKF; still model/parameter sensitive [105].	Moderate compute; often more robust than EKF in nonlinear cases [105].
ANFIS	Training-dependent (multi-sensor fusion; typically IMU/GNSS and/or other localization sensors)	Improved position estimate	Combines fuzzy decision-making flexibility with neural learning and can continuously improve position estimation; reported as more accurate than EKF [106].	Requires large-scale data collection to generalize across different localization conditions [106].	Training cost high; inference can be real-time depending on model size [106].
LSTM-based fusion	IMU + GPS (continuous fusion)	Drift-reduced position estimate	Deep learning can reduce drift and model nonlinearities effectively; LSTM learns temporal patterns and is suitable when past states affect future behavior [107].	Requires representative training data; risk of reduced performance outside the training distribution [107].	Training-heavy; inference can be real-time on suitable embedded hardware [107].

## Data Availability

No new data were created or analyzed in this study. Data sharing is not applicable to this article.
